# Supplementary material for: Lecanicilliums A–F, Thiodiketopiperazine-Class Alkaloids from a Mangrove Sediment-Derived Fungus Lecanicillium kalimantanense
Source: Mar Drugs. 2023 Oct 31;21(11):575. doi: 10.3390/md21110575 (PMC10672332; doi:10.3390/md21110575)

Lecanicilliums A–F, Thiodiketopiperazine-Class Alkaloids from a Mangrove Sediment-Derived Fungus *Lecanicillium kalimantanense*

Lin-Fang Zhong [1,2], Juan Ling [1], Lian-Xiang Luo [3], Chang-Nian Yang [3], Xiao Liang [1] and Shu-Hua Qi [1,*]

[1] CAS Key Laboratory of Tropical Marine Bio-Resources and Ecology, Guangdong Key Laboratory of Marine Materia Medica, South China Sea Institute of Oceanology, Chinese Academy of Sciences, Guangzhou 510301, China; 18851107875@163.com (L.-F.Z.); lingjuan@scsio.ac.cn (J.L.); liangxiao@scsio.ac.cn (X.L.)

[2] University of Chinese Academy of Sciences, Beijing 100049, China

[3] The Marine Biomedical Research Institute, Guangdong Medical University, Zhanjiang 524023, China; luolianxiang321@gdmu.edu.cn (L.-X.L.); nicholasyeung@gdmu.edu.cn (C.-N.Y.)

* Correspondence: shuhuaqi@scsio.ac.cn; Tel.: +86-208-9022-112; Fax: +86-208-4458-964

CONTENT

Figure S1 ^{1}H NMR spectrum of compound **1** in DMSO-d_6..4
Figure S2 ^{13}C NMR spectrum of compound **1** in DMSO-d_6 5
Figure S3 ^{1}H-^{1}H COSY spectrum of compound **1** in DMSO-d_6.................................5
Figure S4 HSQC spectrum of compound **1** in DMSO-d_6 6
Figure S5 HMBC spectrum of compound **1** in DMSO-d_6 7
Figure S6 NOESY spectrum of compound **1** in DMSO-d_6...8
Figure S7 HR-ESIMS spectrum of compound **1** 8
Figure S8 IR spectrum of compound **1** 9
Figure S9 UV spectrum of compound **1** 10
Figure S10 ^{1}H NMR spectrum of compound **2** in DMSO-d_6 11
Figure S11 ^{13}C NMR spectrum of compound **2** in DMSO-d_6....................................11
Figure S12 ^{1}H-^{1}H COSY spectrum of compound **2** in DMSO-d_6.............................12
Figure S13 HSQC spectrum of compound **2** in DMSO-d_6 12
Figure S14 HMBC spectrum of compound **2** in DMSO-d_6 13
Figure S15 NOESY spectrum of compound **2** in DMSO-d_6.......................................14
Figure S16 HR-ESIMS spectrum of compound **2** 15
Figure S17 IR spectrum of compound **2** 15
Figure S18 UV spectrum of compound **2** 16

Figure S19 ^{1}H NMR spectrum of compound **3** in DMSO-d_6 17
Figure S20 ^{13}C NMR spectrum of compound **3** in DMSO-d_6 18
Figure S21 ^{1}H-^{1}H COSY spectrum of compound **3** in DMSO-d_6.............................18
Figure S22 HSQC spectrum of compound **3 i**n DMSO-d_6 19
Figure S23 HMBC spectrum of compound **3** in DMSO-d_6..20
Figure S24 NOESY spectrum of compound **3** in DMSO-d_6.......................................21
Figure S25 HR-ESIMS spectrum of compound **3** 21
Figure S26 IR spectrum of compound **3**..22
Figure S27 UV spectrum of compound **3** 23
Figure S28 ^{1}H NMR spectrum of compound **4** in DMSO-d_6 24
Figure S29 ^{13}C NMR spectrum of compound **4** in DMSO-d_6 24
Figure S30 ^{1}H-^{1}H COSY spectrum of compound **4** in DMSO-d_6.............................25
Figure S31 HSQC spectrum of compound **4** in DMSO-d_6 25
Figure S32 HMBC spectrum of compound **4** in DMSO-d_6..26
Figure S33 NOESY spectrum of compound **4** in DMSO-d_6.......................................27
Figure S34 HR-ESIMS spectrum of compound **4** 27
Figure S35 IR spectrum of compound **4**..28
Figure S36 UV spectrum of compound **4** 40
Figure S37 ^{1}H NMR spectrum of compound **5** in DMSO-d_6 30
Figure S38 ^{13}C NMR spectrum of compound **5** in DMSO-d_6 30
Figure S39 ^{1}H-^{1}H COSYspectrum of compound **5** in DMSO-d_6 31
Figure S40 HSQC spectrum of compound **5** in DMSO-d_6 31
Figure S41 HMBC spectrum of compound **5** in DMSO-d_6..32
Figure S42 NOESY spectrum of compound **5** in DMSO-d_6.......................................33
Figure S43 HR-ESIMS spectrum of compound **5** 33
Figure S44 IR spectrum of compound **5**..34
Figure S45 UV spectrum of compound **5** 35
Figure S46 ^{1}H NMR spectrum of compound **6** in DMSO-d_6 36
Figure S47 ^{13}C NMR spectrum of compound **6** in DMSO-d_6 36
Figure S48 ^{1}H-^{1}H COSYspectrum of compound **6** in DMSO-d_6 37
Figure S49 HSQC spectrum of compound **6** in DMSO-d_6 37
Figure S50 HMBC spectrum of compound **6** in DMSO-d_6..38
Figure S51 NOESY spectrum of compound **6** in DMSO-d_6.......................................39
Figure S52 HR-ESIMS spectrum of compound **6** 40
Figure S53 IR spectrum of compound **6**..41
Figure S54 UV spectrum of compound **6** 42
Figure S55 X-ray crystallographic structure of compound **3**.......................................43
Table S1 Crystal Data and Structure Refinement for Compound **3** 43

Table S2 Relative thermal energies (ΔE), relative free energies (ΔG), and equilibrium populations (P) of low-energy conformers of structures **1-6** in MeOH or MeCN....... 44
Figure S56 Conformations of low-energy conformers of structure **1** in MeOH......... 45
Figure S57 Conformations of low-energy conformers of structure **2** in MeOH......... 45
Figure S58 Conformations of low-energy conformers of structure **3** in CH_3CN 46
Figure S59 Conformations of low-energy conformers of structure **4** in MeOH......... 47
Figure S60 Conformations of low-energy conformers of structure **5** in MeOH......... 48
Figure S61 Conformations of low-energy conformers of structure **6** in MeOH......... 49
Figure S62 CD spectrum of **3** in CH_3CN... 49
Figure S63 CD spectra of **1**, **2**, **4-6** in MeOH 50
Figure S64 Key HMBC and COSY correlations of compounds **5-6**…………………50
Figure S65 Key NOESY correlations of compounds **5-6**……………………………50
Figure S66 The proposed biosynthetic pathway for compound **1**……………………51

Figure S1 ^{1}H NMR spectrum of compound **1** in DMSO-d_6

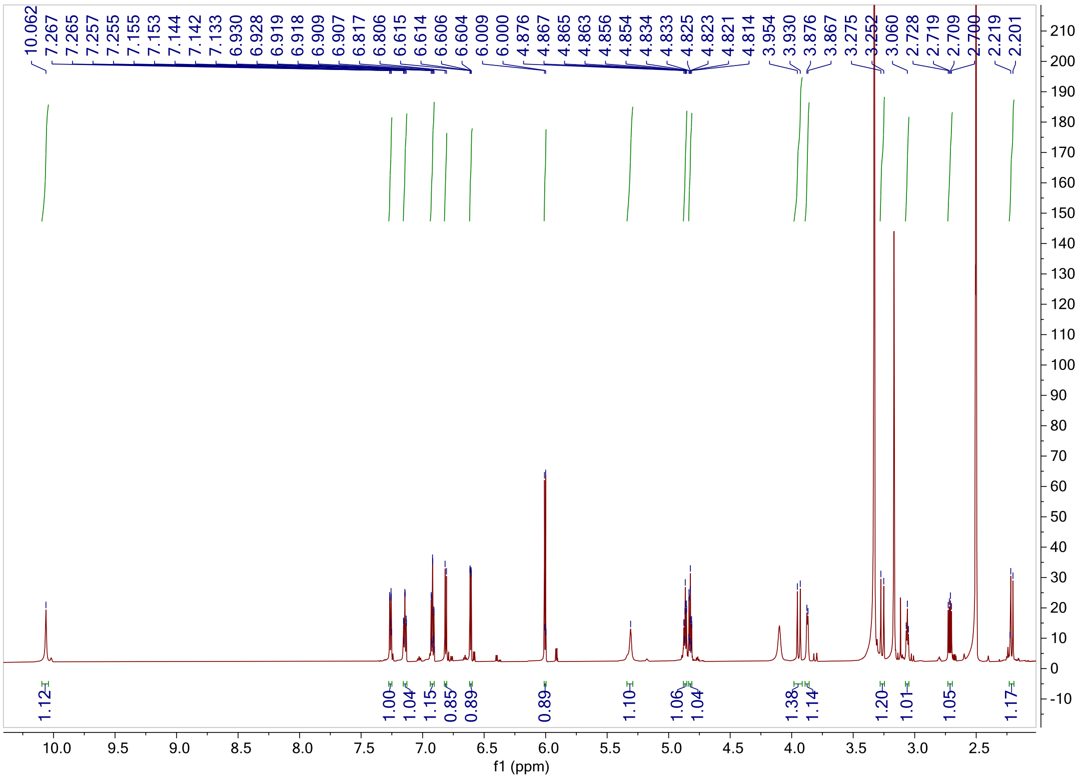

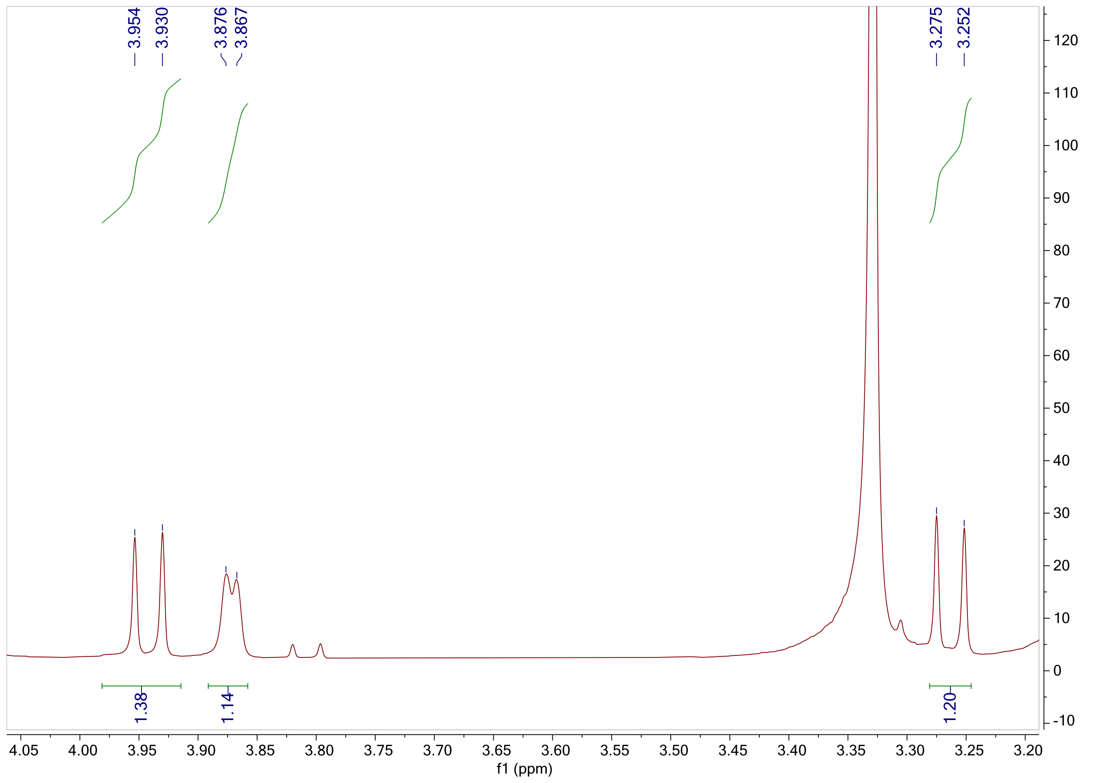

Figure S2 ^{13}C NMR spectrum of compound **1** in DMSO-d_6

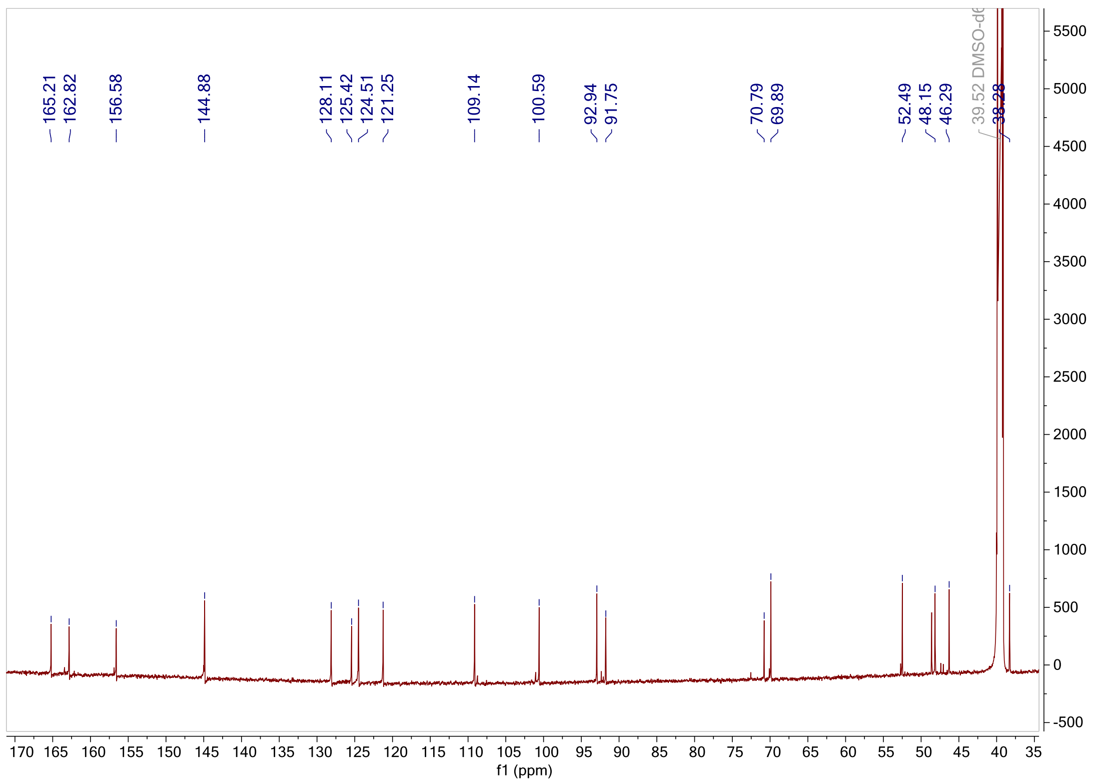

Figure S3 ^{1}H-^{1}H COSY spectrum of compound **1** in DMSO-d_6

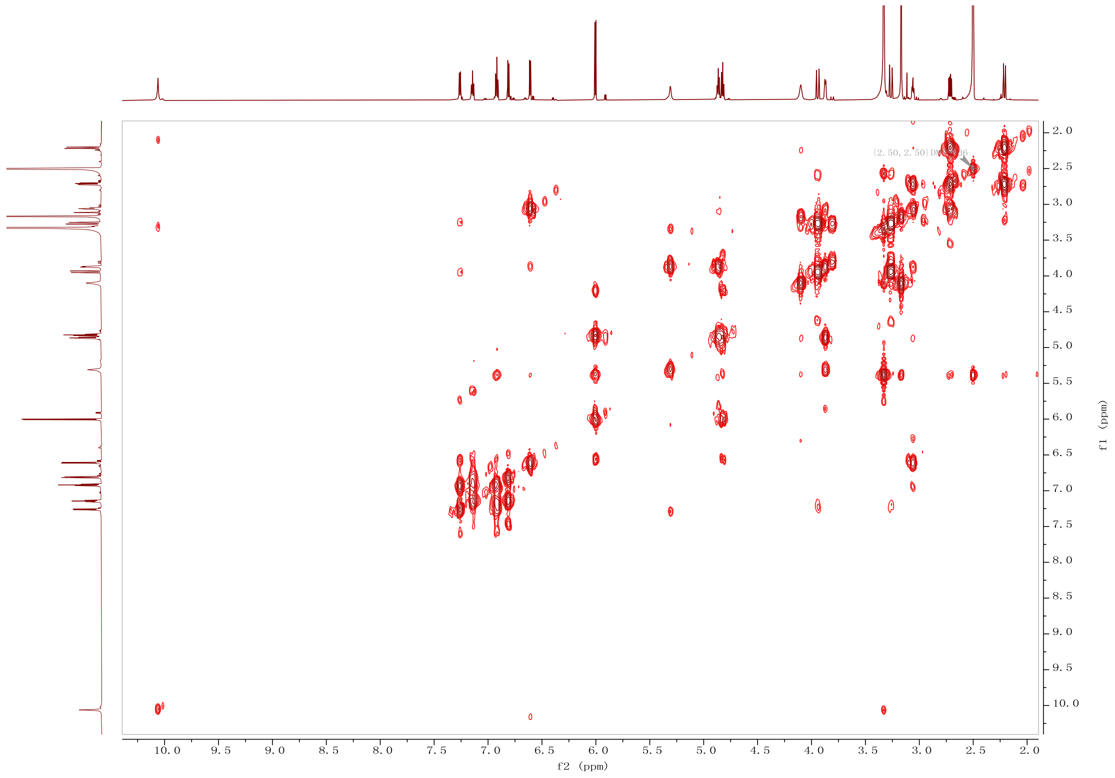

Figure S4 HSQC spectrum of compound **1** in DMSO-d_6

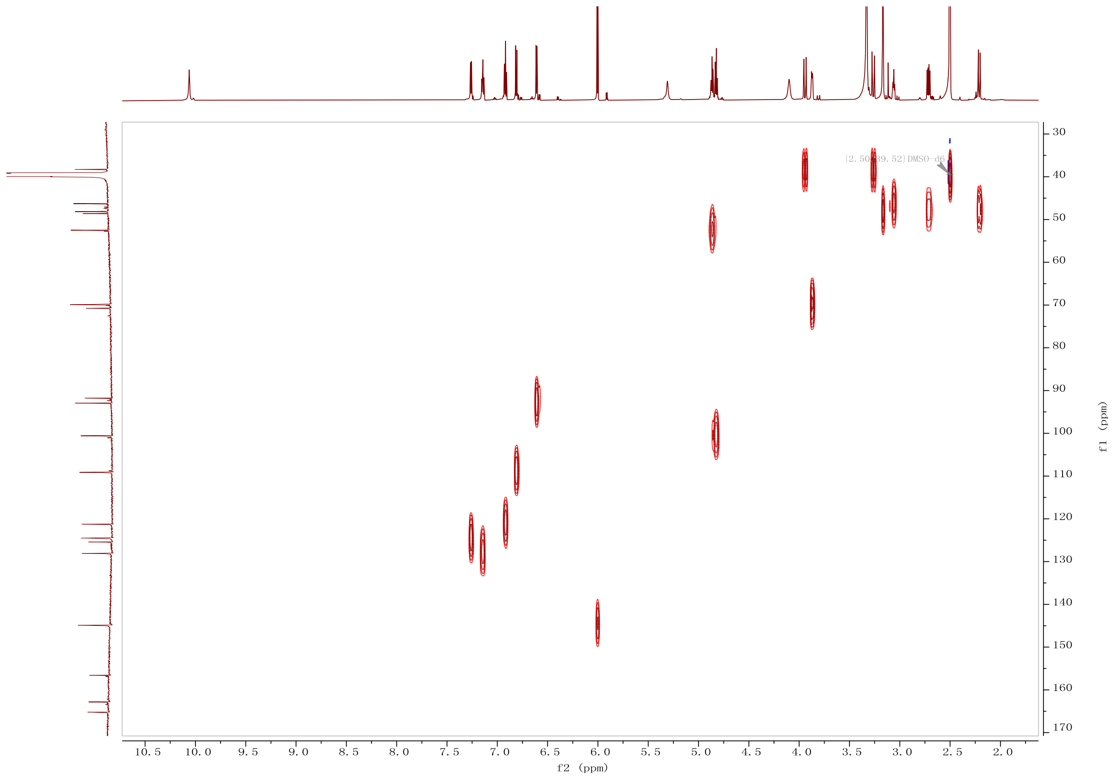

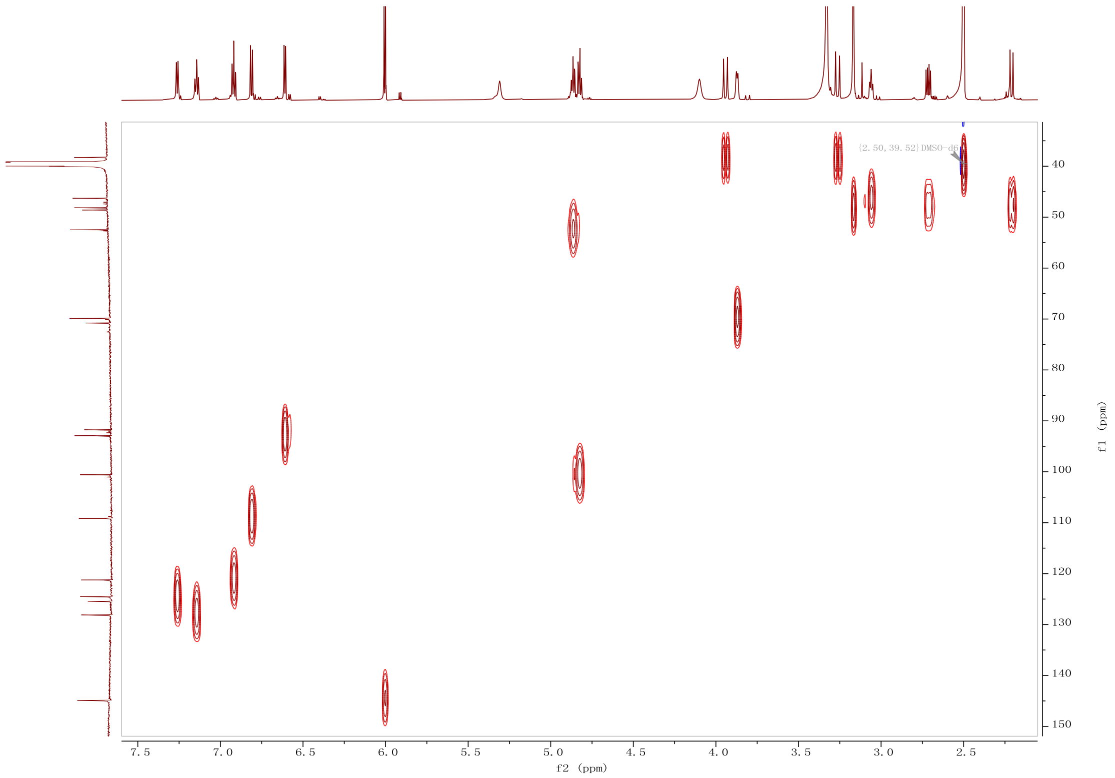

Figure S5 HMBC spectrum of compound **1** in DMSO-d_6

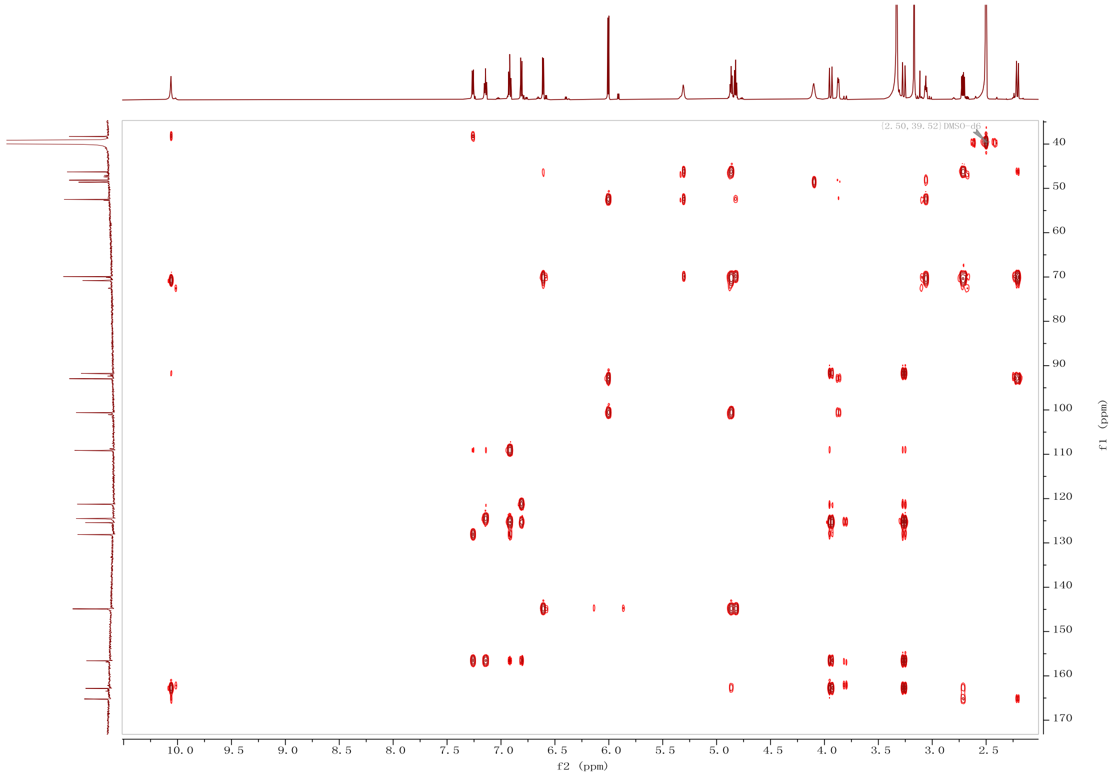

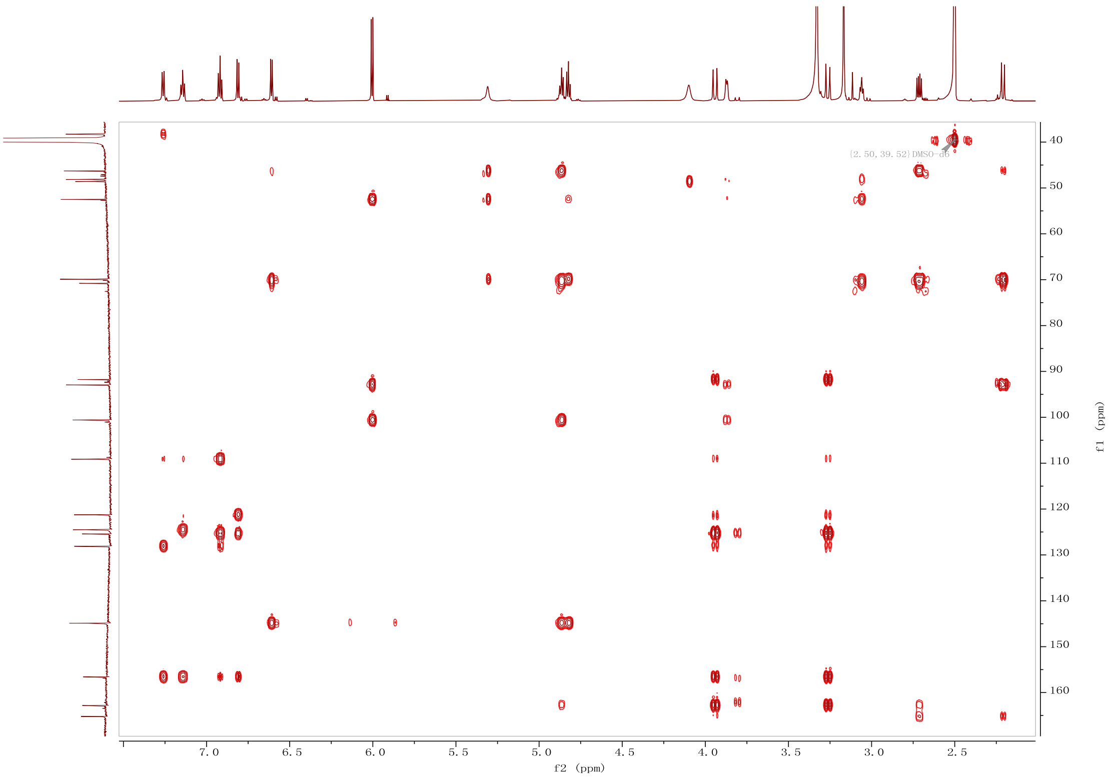

Figure S6 NOESY spectrum of compound **1** in DMSO-d_6

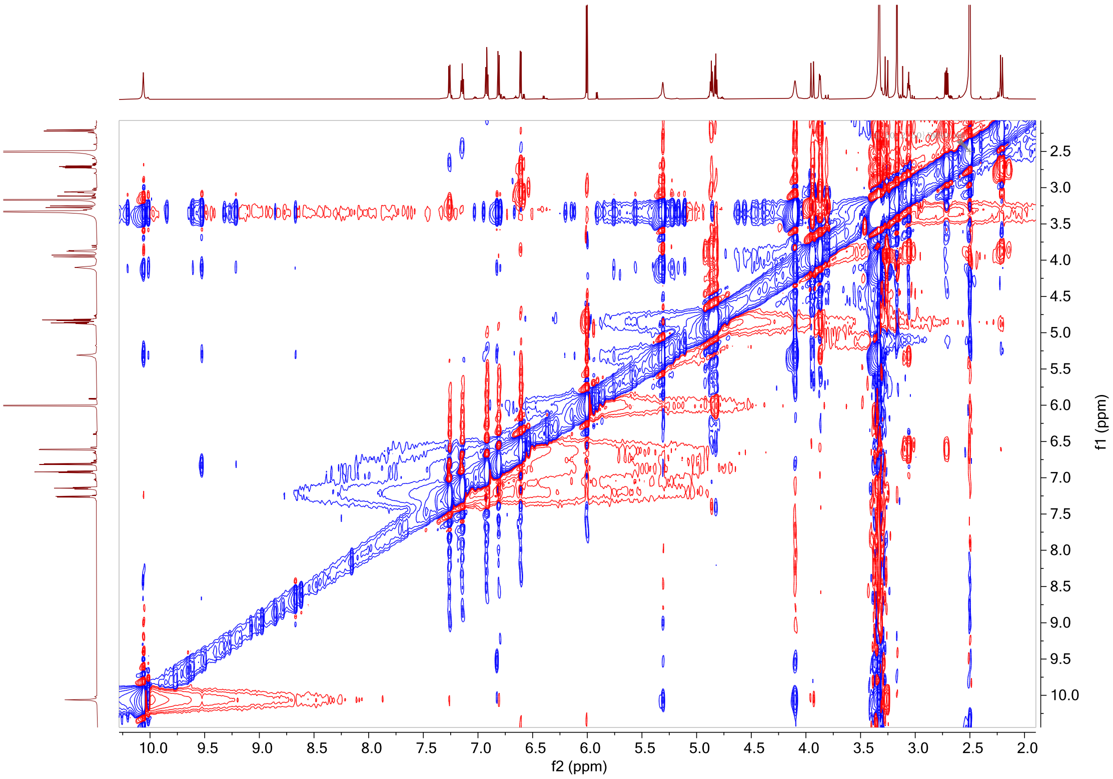

Figure S7 HR-ESIMS spectrum of compound **1**

Mass Spectrum SmartFormula Report

**Analysis Info**			
Analysis Name	D:\Data\MS\data\202304\zhonglinfang_QZLF-L-35_pos_51_01_14978.d	Acquisition Date	5/5/2023 4:27:52 PM
Method	LC_Direct Infusion_pos_70-500mz.m	Operator	SCSIO
Sample Name	zhonglinfang_QZLF-L-35_pos	Instrument	maXis 255552.00029
Comment			

**Acquisition Parameter**					
Source Type	ESI	Ion Polarity	Positive	Set Nebulizer	0.4 Bar
Focus	Active	Set Capillary	4500 V	Set Dry Heater	180 °C
Scan Begin	100 m/z	Set End Plate Offset	-500 V	Set Dry Gas	4.0 l/min
Scan End	1500 m/z	Set Charging Voltage	0 V	Set Divert Valve	Waste
		Set Corona	0 nA	Set APCI Heater	0 °C

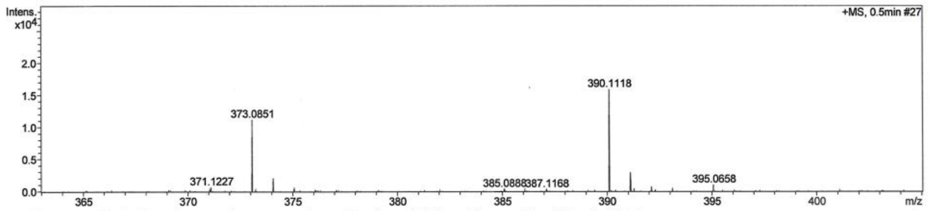

Meas. m/z	#	Ion Formula	Score	m/z	err [ppm]	err [mDa]	mSigma	rdb	e⁻ Conf	N-Rule
373.0851	1	C18H17N2O5S	100.00	373.0853	-0.5	-0.2	22.1	11.5	even	ok
390.1118	1	C18H20N3O5S	100.00	390.1118	0.1	0.0	21.5	10.5	even	ok
395.0658	1	C18H16N2NaO5S	100.00	395.0672	3.7	1.5	15.1	11.5	even	ok

zhonglinfang_QZLF-L-35_pos_51_01_14978.d

Bruker Compass DataAnalysis 4.1 printed: 5/5/2023 4:32:00 PM by: SCSIO Page 1 of 1

Figure S8 IR spectrum of compound **1**

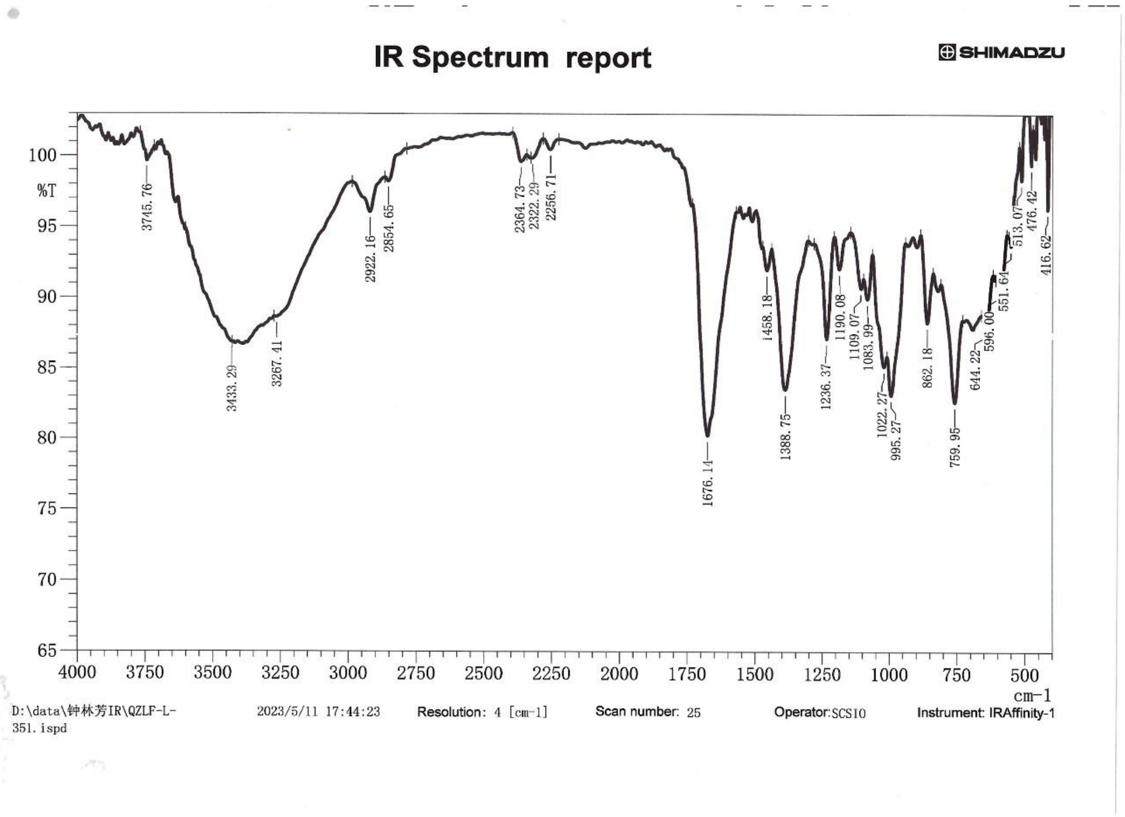

Figure S9 UV spectrum of compound **1**

光谱峰值检测报告

2023/05/11 16:59:25

数据集: QZLF-L-35 - RawData

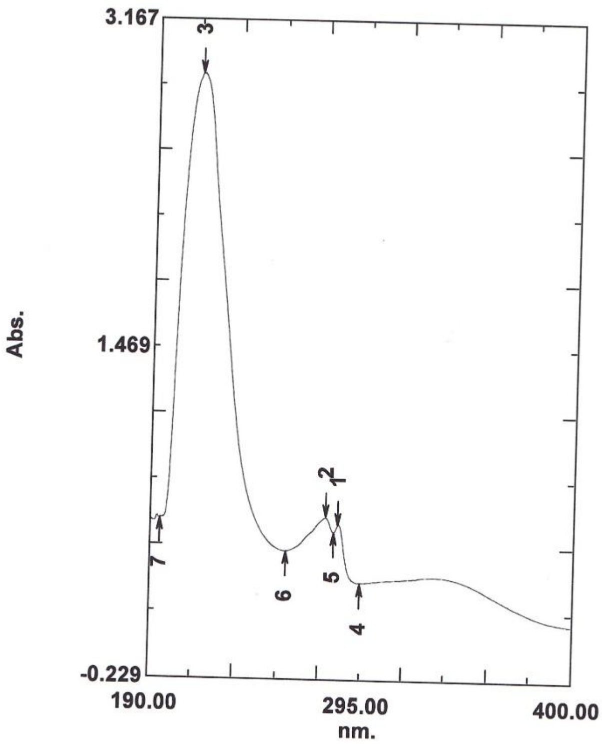

[测定属性]
波长范围(nm): 190.00 到 400.00
扫描速度: 中速
采样间隔: 0.2
自动采样间隔: 启用
扫描模式: 单个

[仪器属性]
仪器类型: UV-2600 系列
测定方式: 吸收值
狭缝宽: 2.0
积分时间: 0.1 秒.
光源转换波长: 323.0 nm
检测器单元: 直接
S/R 转换: 标准
阶梯校正: OFF

[附件属性]
附件: 无

[数据处理参数]
阈值: 0.0500000
点: 4
内插: 停用
平均: 停用

[样品准备属性]
重量:
体积:
稀释:
光程长:
附加信息:

No.	P/V	波长(nm)	Abs.	描述
1	↑	283.20	0.560	
2	↑	276.60	0.596	
3	↑	211.80	2.884	
4	↓	294.40	0.261	
5	↓	280.80	0.520	
6	↓	257.20	0.423	
7	↓	194.40	0.585	

页 1 / 1

Figure S10 ^{1}H NMR spectrum of compound **2** in DMSO-d_6

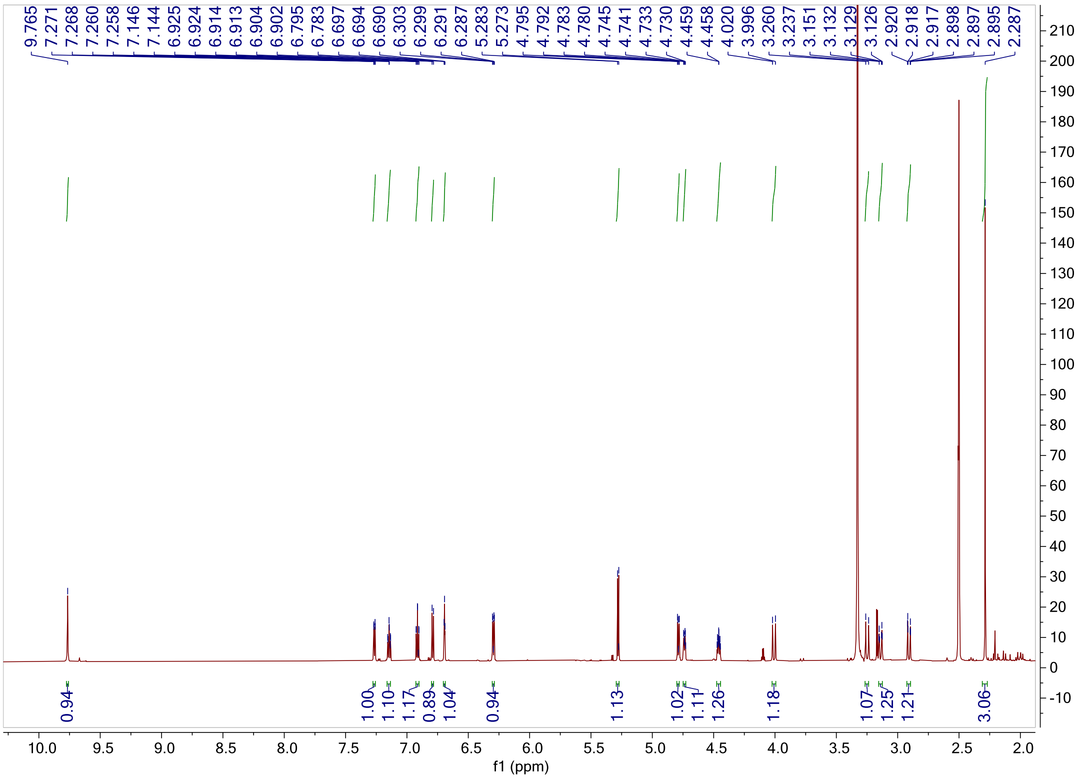

Figure S11 ^{13}C NMR spectrum of compound **2** in DMSO-d_6

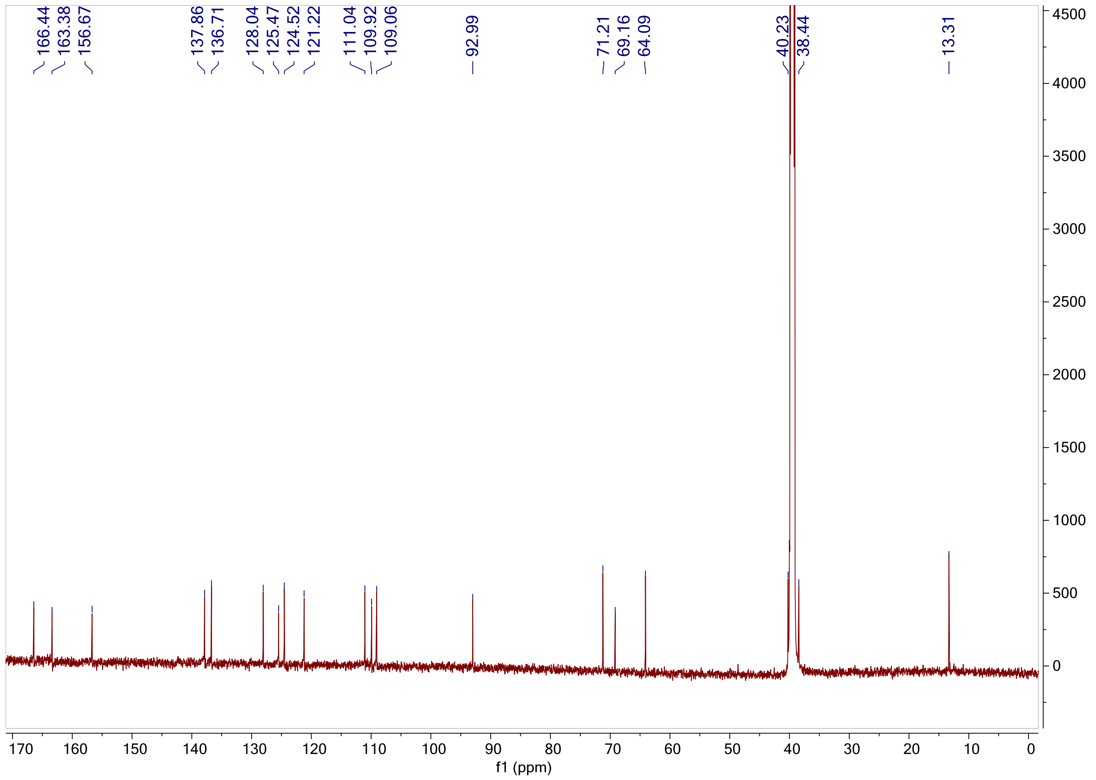

Figure S12 ^{1}H-^{1}H COSY spectrum of compound **2** in DMSO-d_6

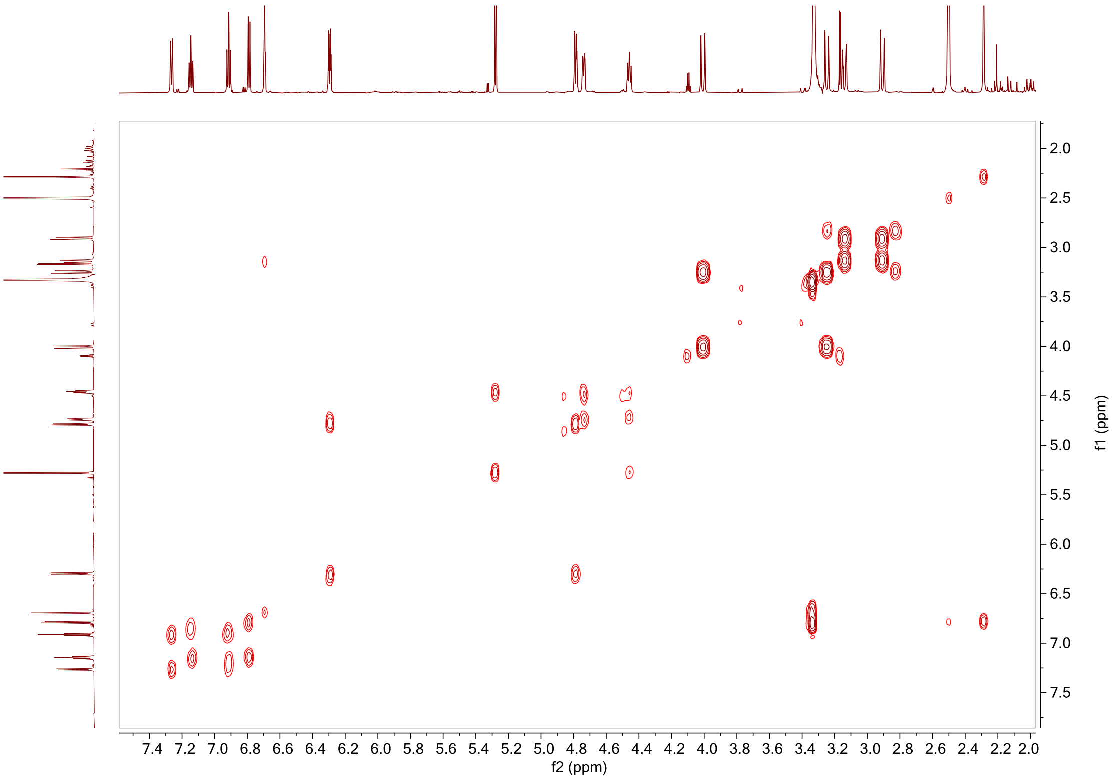

Figure S13 HSQC spectrum of compound **2** in DMSO-d_6

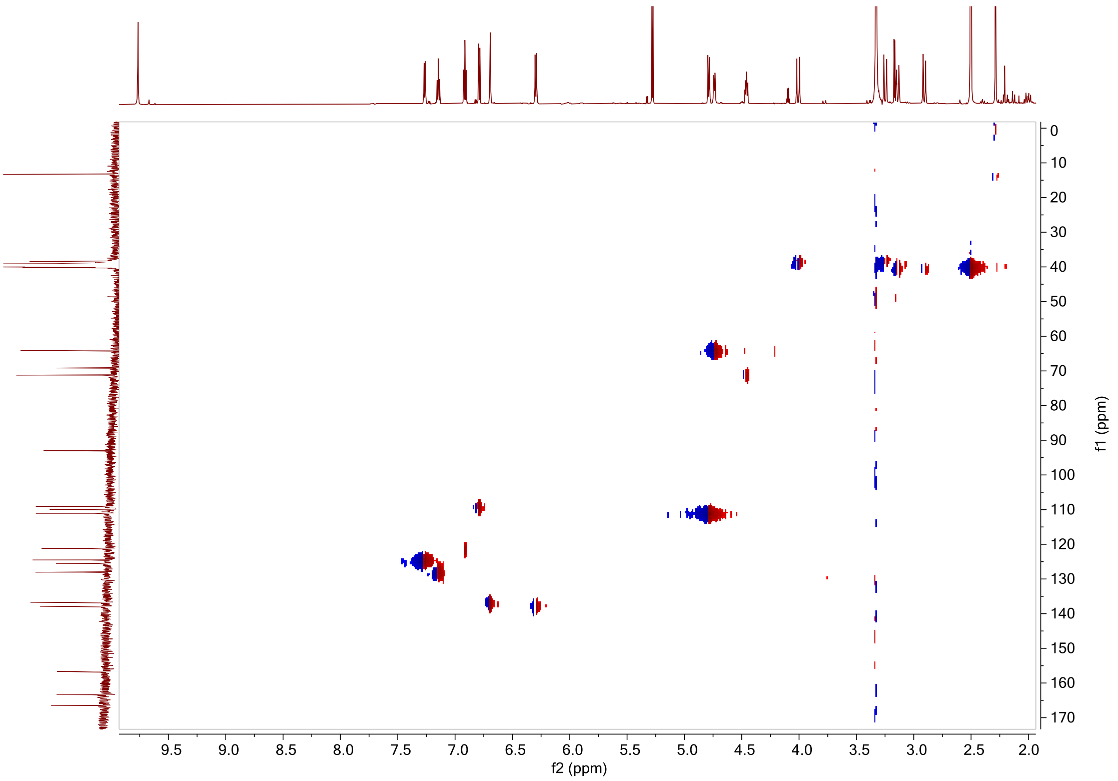

Figure S14 HMBC spectrum of compound **2** in DMSO-d_6

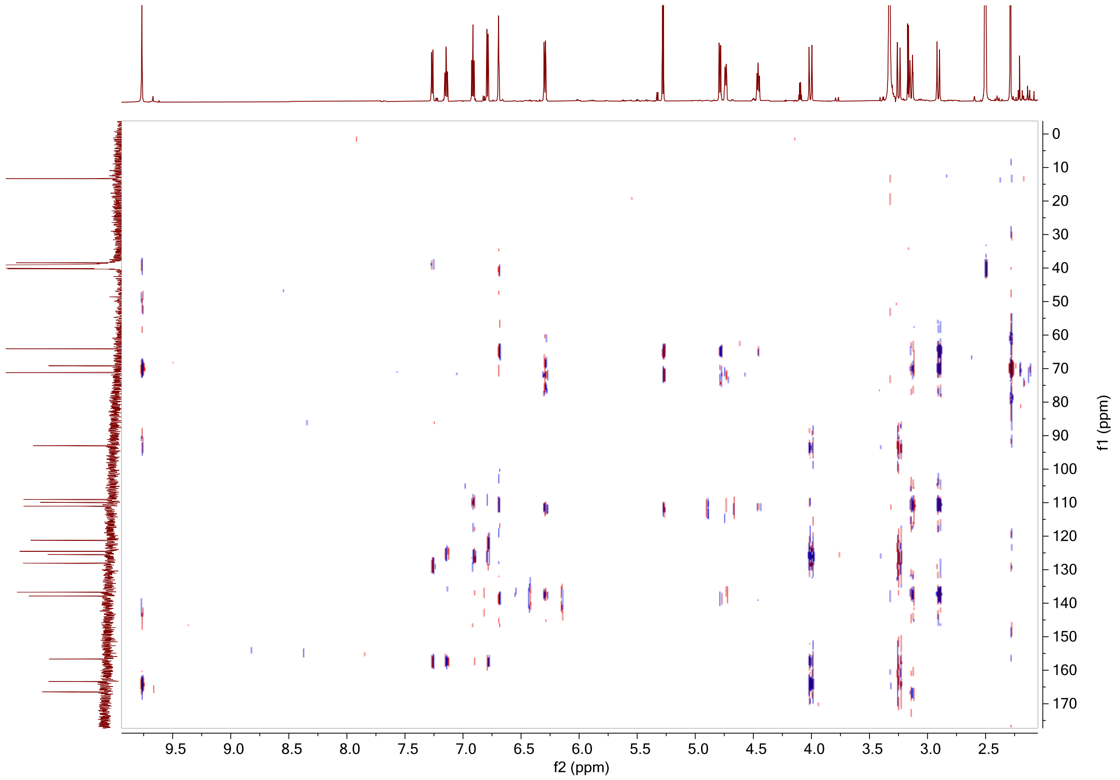

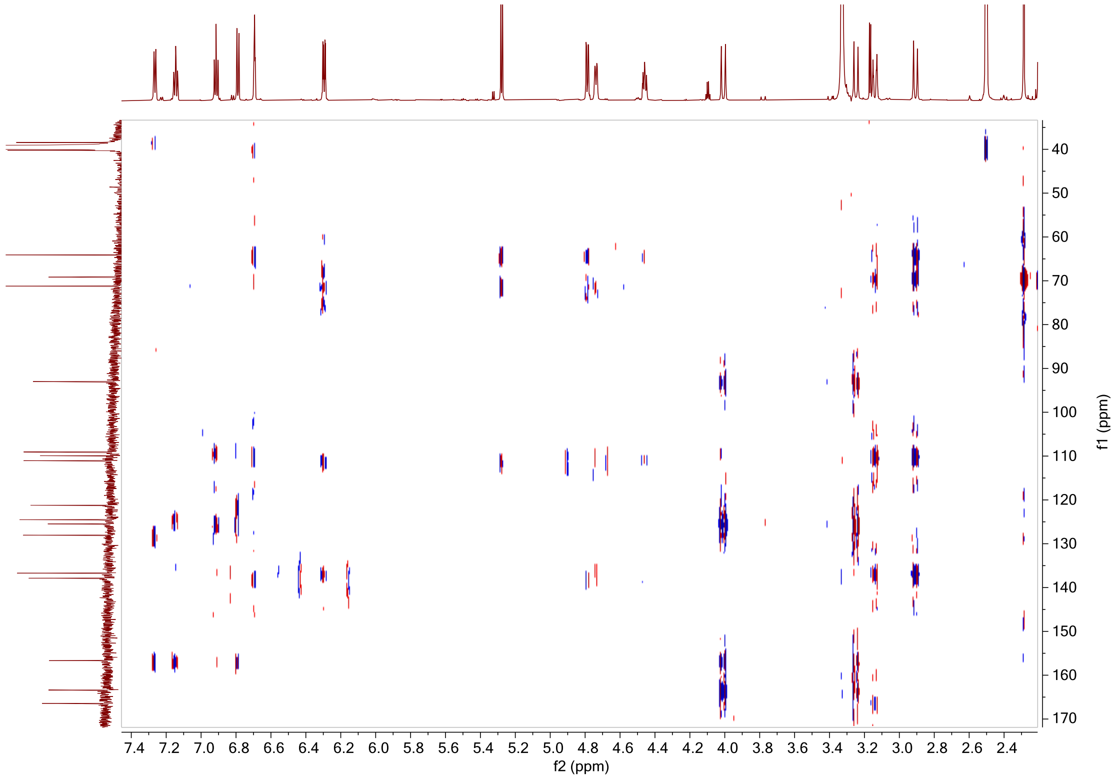

Figure S15 NOESY spectrum of compound **2** in DMSO-d_6

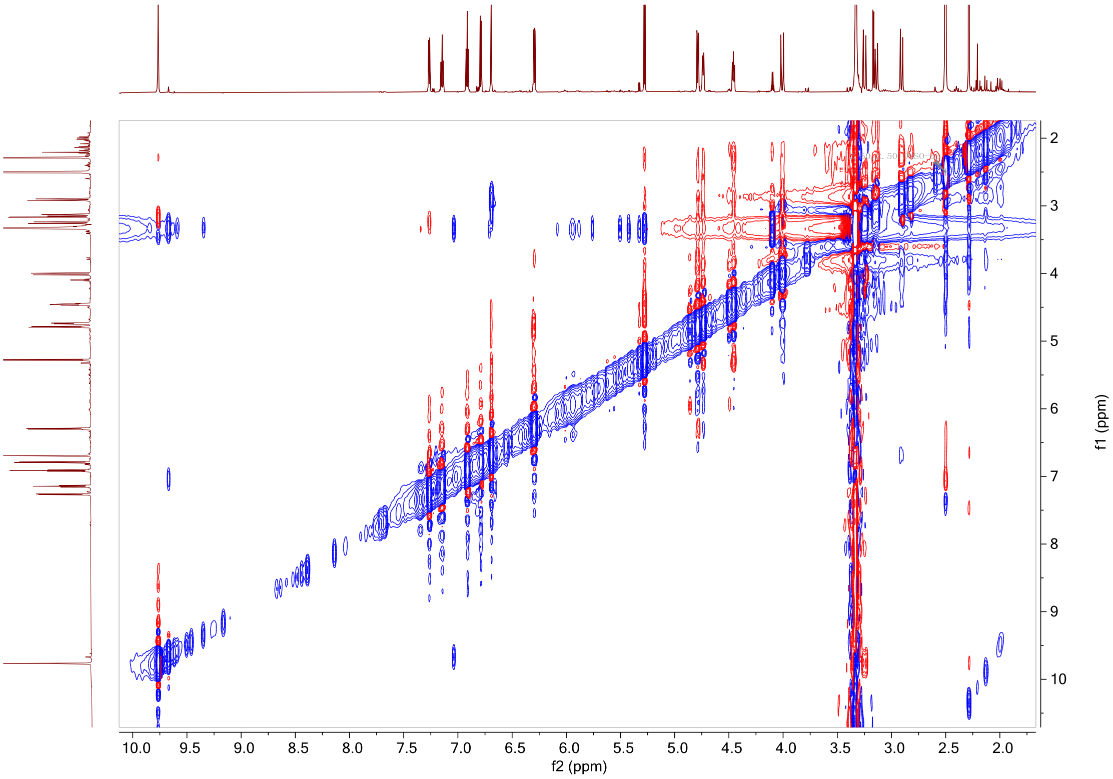

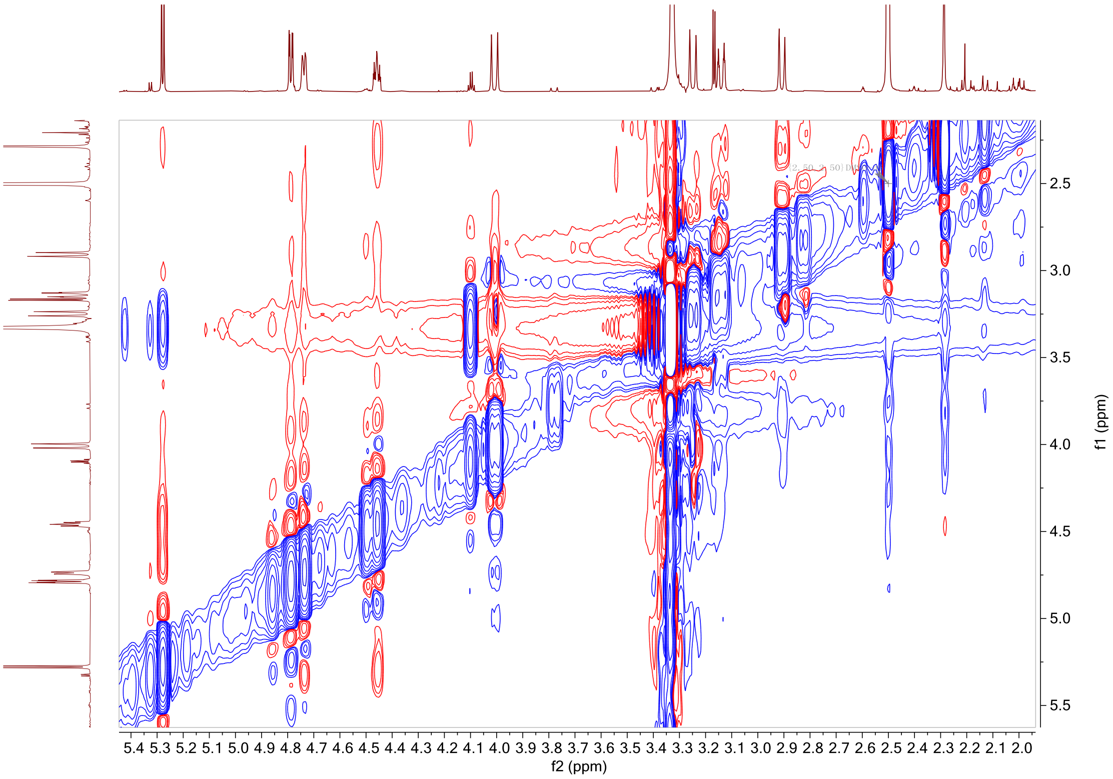

Figure S16 HR-ESIMS spectrum of compound **2**

Mass Spectrum SmartFormula Report

Analysis Info

Analysis Name	D:\Data\MS\data\202212\zhonglinfangQZLF-L-15_pos_55_01_14154.d	Acquisition Date	12/13/2022 5:32:31 PM
Method	LC_Direct Infusion_pos_70-500mz.m	Operator	SCSIO
Sample Name	zhonglinfangQZLF-L-15_pos	Instrument	maXis 255552.00029
Comment			

Acquisition Parameter

Source Type	ESI	Ion Polarity	Positive	Set Nebulizer	0.4 Bar
Focus	Active	Set Capillary	4500 V	Set Dry Heater	180 °C
Scan Begin	100 m/z	Set End Plate Offset	-500 V	Set Dry Gas	4.0 l/min
Scan End	1500 m/z	Set Charging Voltage	0 V	Set Divert Valve	Waste
		Set Corona	0 nA	Set APCI Heater	0 °C

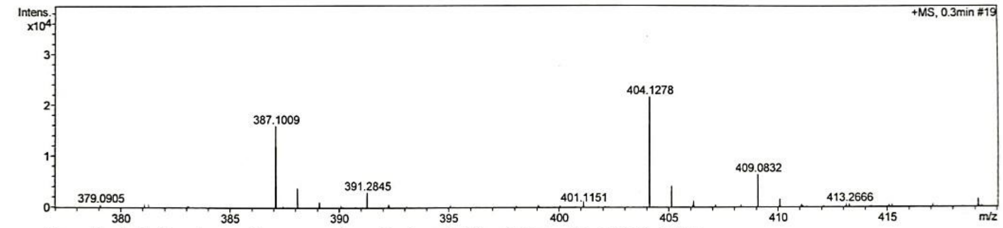

Meas. m/z	#	Ion Formula	Score	m/z	err [ppm]	err [mDa]	mSigma	rdb	e⁻ Conf	N-Rule
387.1009	1	C19H19N2O5S	100.00	387.1009	0.1	0.0	10.3	11.5	even	ok
404.1278	1	C19H22N3O5S	100.00	404.1275	0.9	0.4	25.6	10.5	even	ok
409.0832	1	C19H18N2NaO5S	100.00	409.0829	-0.8	-0.3	12.8	11.5	even	ok

zhonglinfangQZLF-L-15_pos_55_01_14154.d

Bruker Compass DataAnalysis 4.1 printed: 12/13/2022 5:38:20 PM by: SCSIO Page 1 of 1

Figure S17 IR spectrum of compound **2**

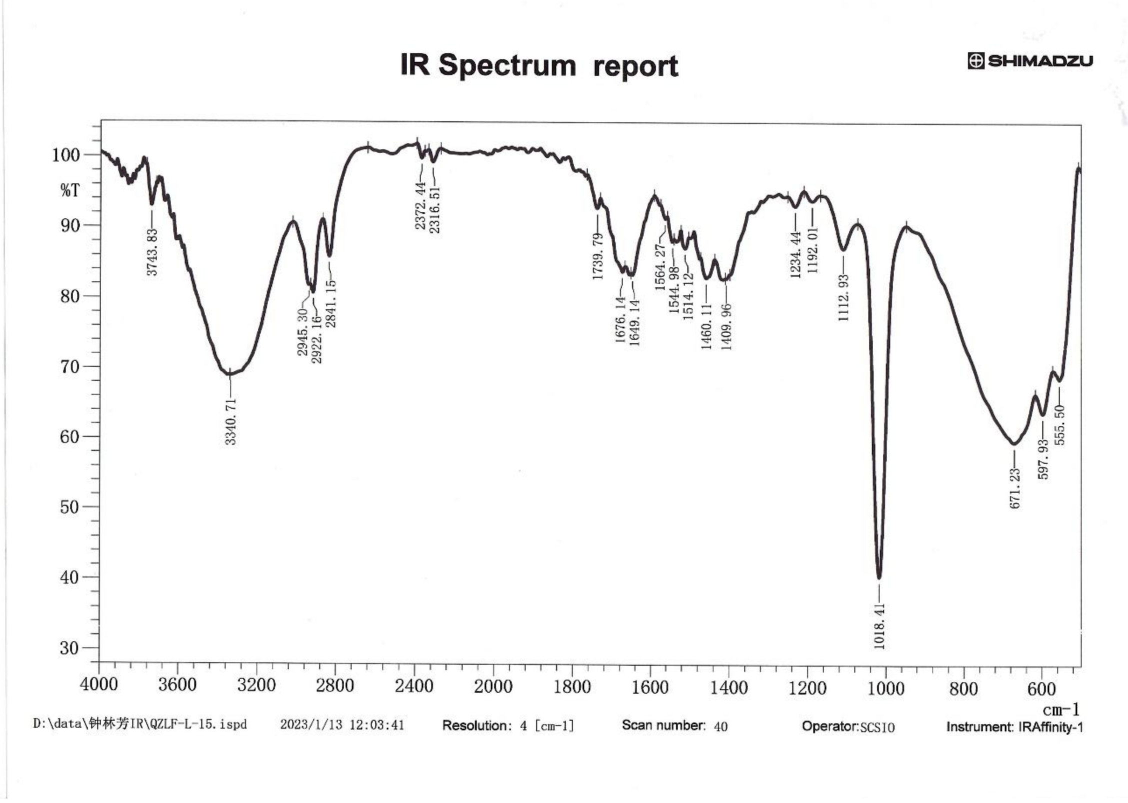

Figure S18 UV spectrum of compound **2**

光谱峰值检测报告

2023/01/12 13:03:44

数据集: QZLF-L-15 - RawData

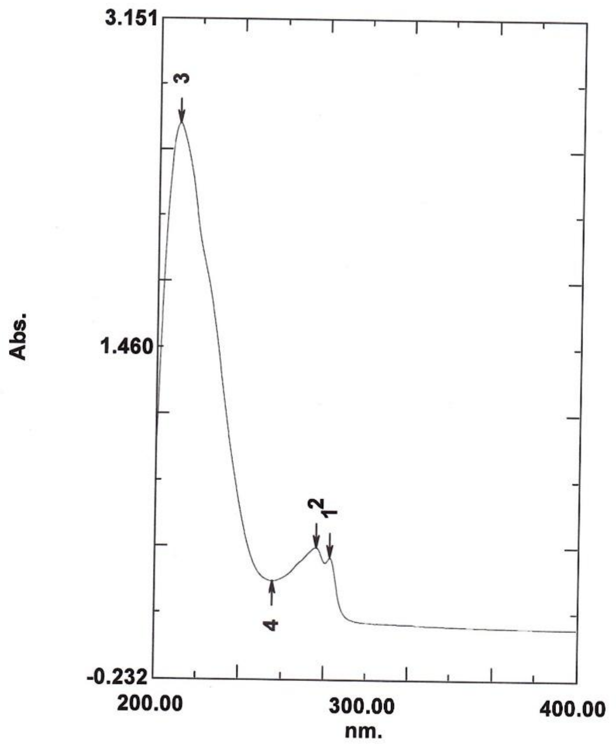

[测定属性]
波长范围(nm): 200.00 到 400.00
扫描速度: 中速
采样间隔: 0.2
自动采样间隔: 启用
扫描模式: 单个

[仪器属性]
仪器类型: UV-2600 系列
测定方式: 吸收值
狭缝宽: 2.0
积分时间: 0.1 秒.
光源转换波长: 323.0 nm
检测器单元: 直接
S/R 转换: 标准
阶梯校正: OFF

[附件属性]
附件: 无

[数据处理参数]
阈值: 0.0500000
点: 4
内插: 停用
平均: 停用

[样品准备属性]
重量:
体积:
稀释:
光程长:
附加信息:

No.	P/V	波长(nm)	Abs.	描述
1	↑	282.80	0.386	
2	↑	276.00	0.439	
3	↑	209.80	2.610	
4	↓	256.00	0.267	

页 1 / 1

Figure S19 ^{1}H NMR spectrum of compound **3** in DMSO-d_6

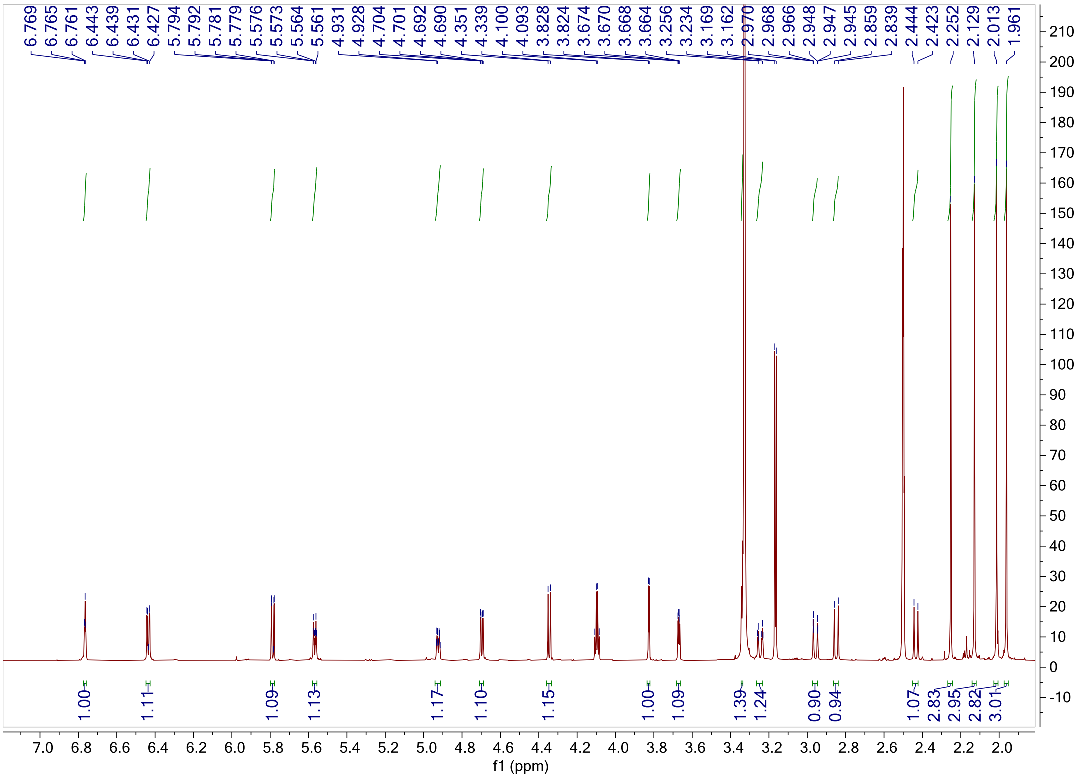

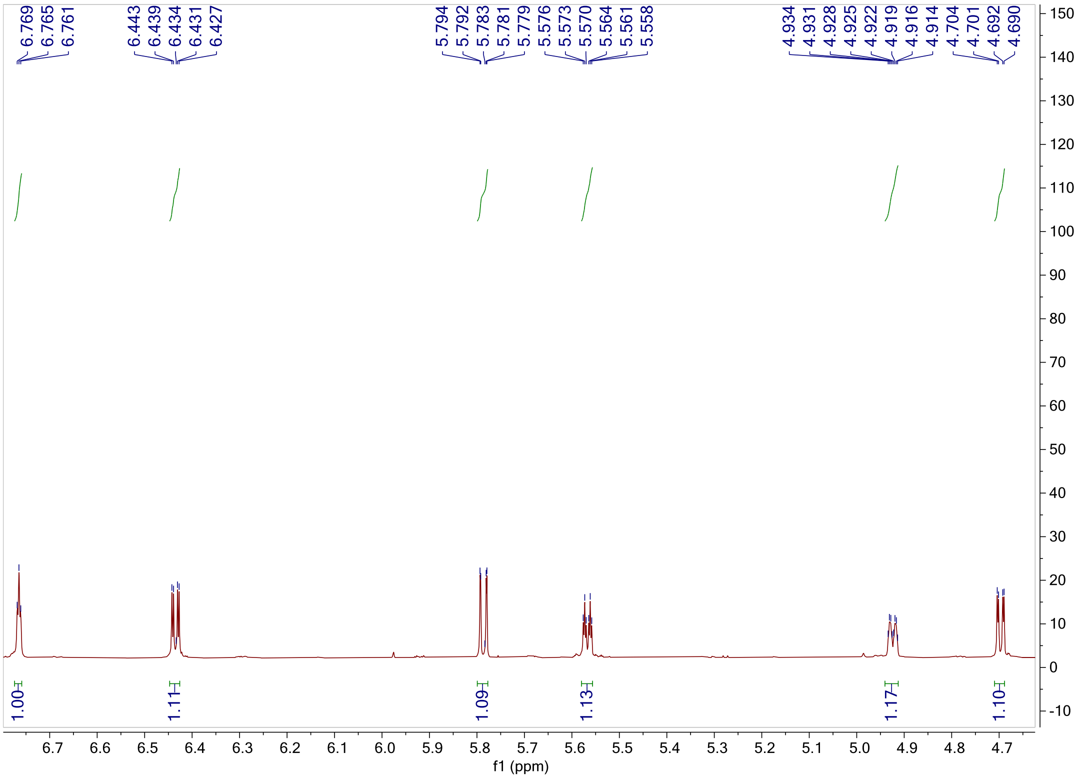

Figure S20 ^{13}C NMR spectrum of compound **3** in DMSO-d_6

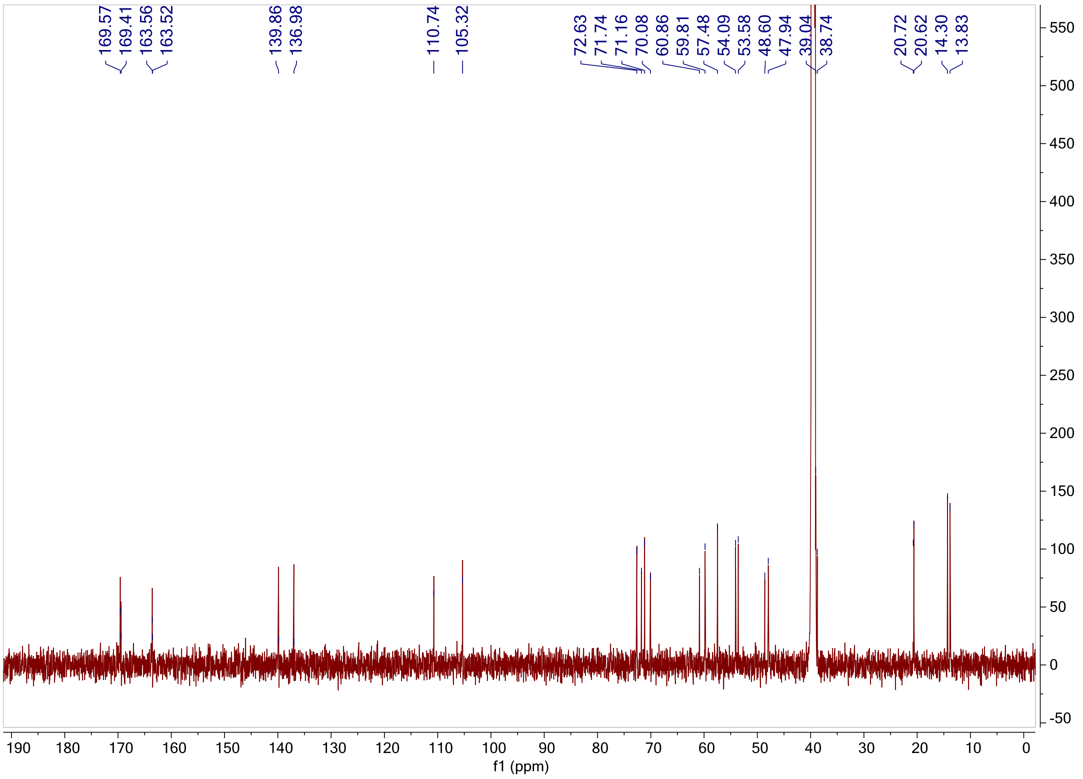

Figure S21 ^{1}H-^{1}H COSY spectrum of compound **3** in DMSO-d_6

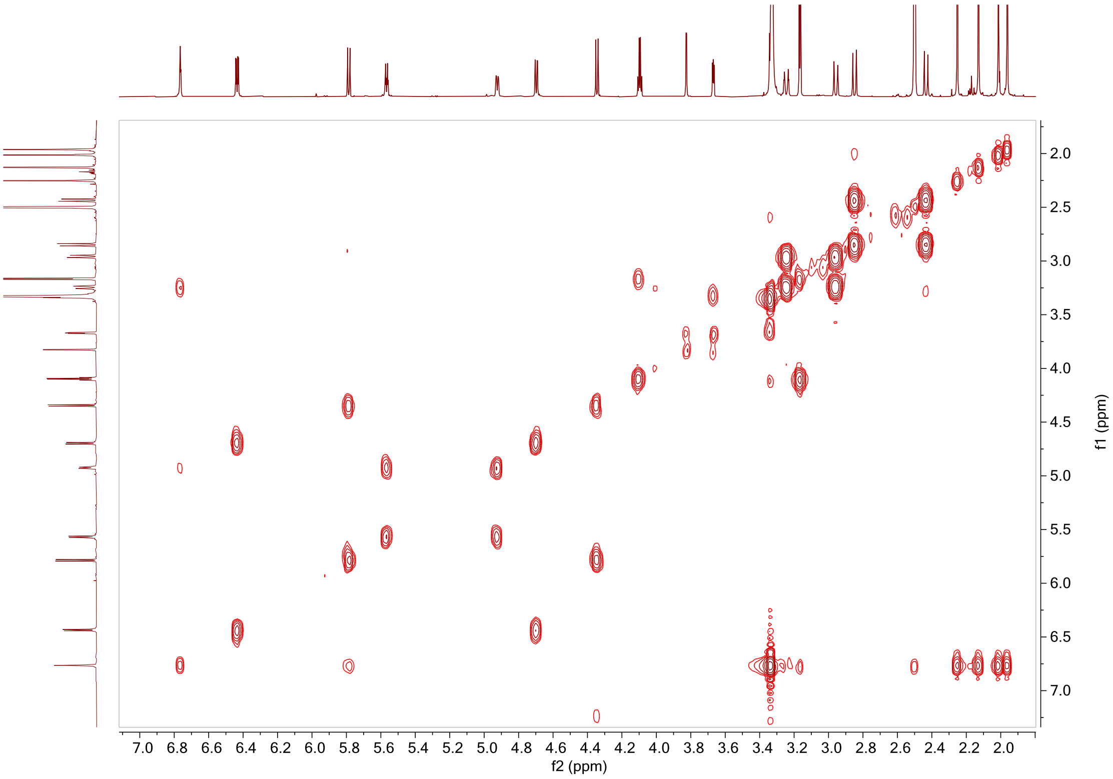

Figure S22 HSQC spectrum of compound **3** in DMSO-d_6

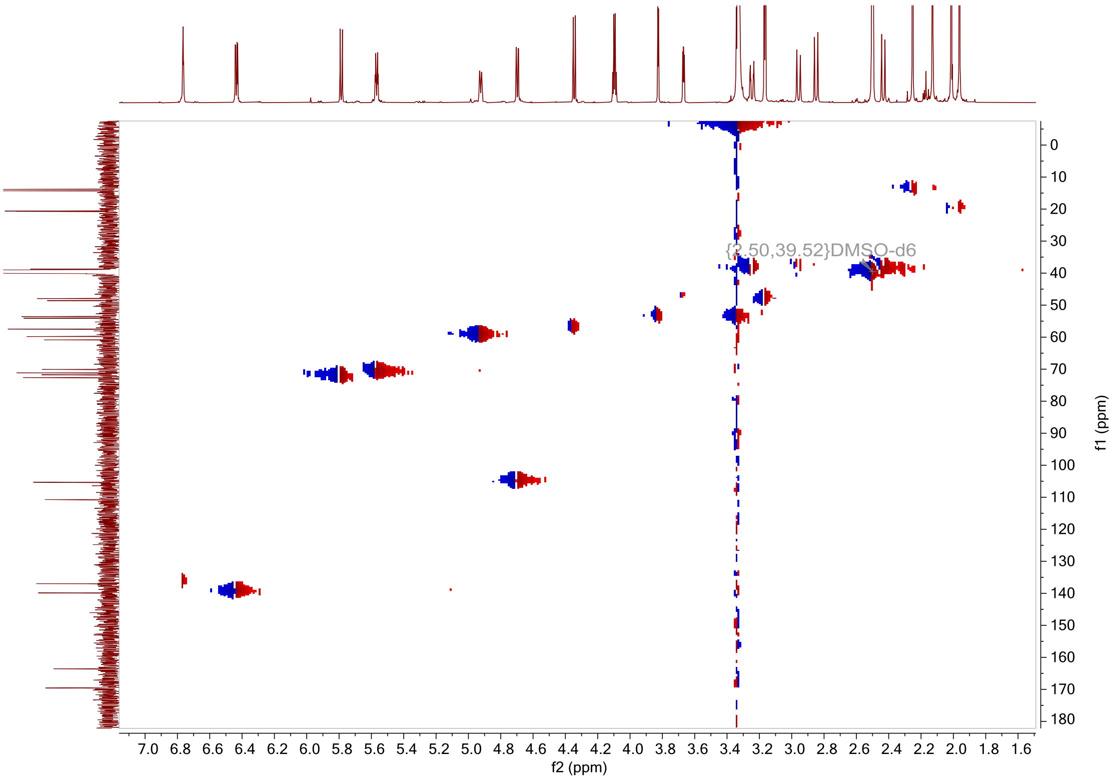

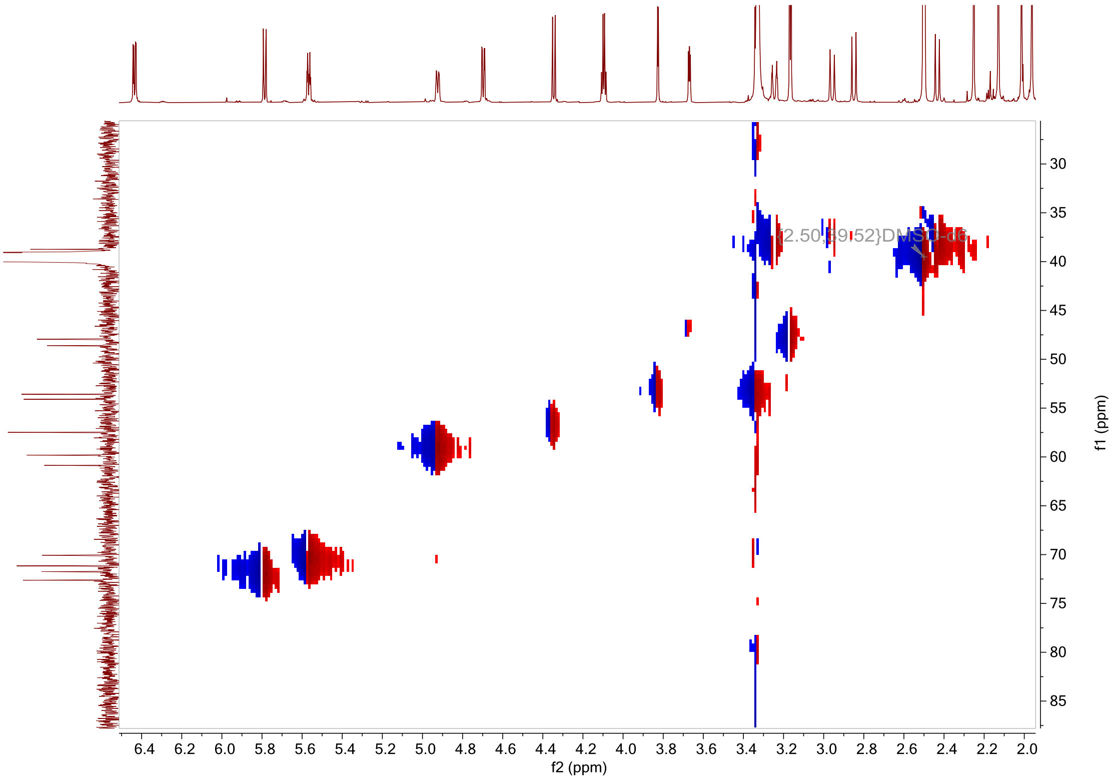

Figure S23 HMBC spectrum of compound **3** in DMSO-d_6

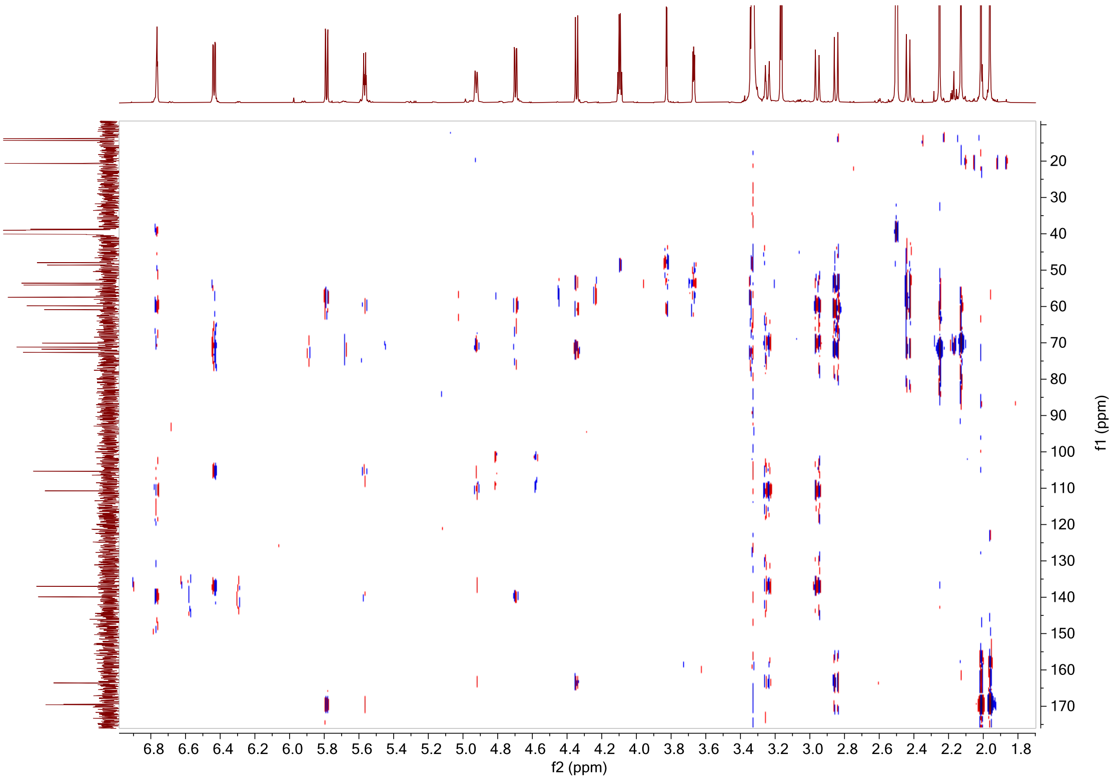

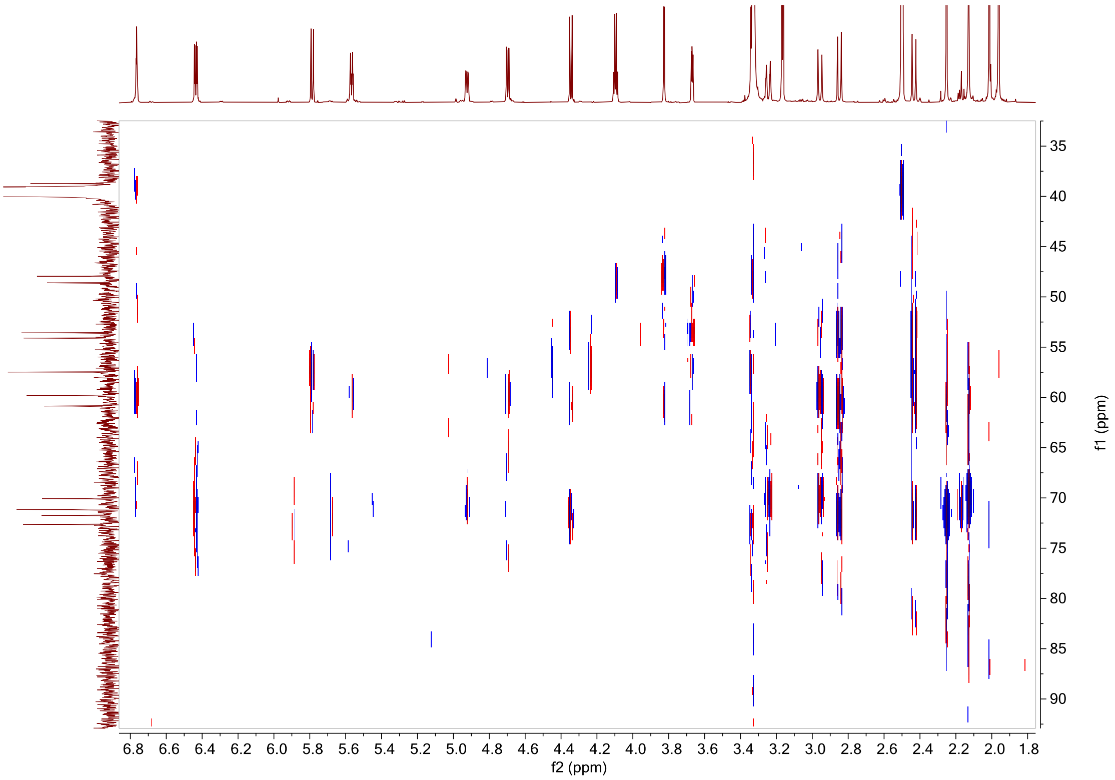

Figure S24 NOESY spectrum of compound **3** in DMSO-d_6

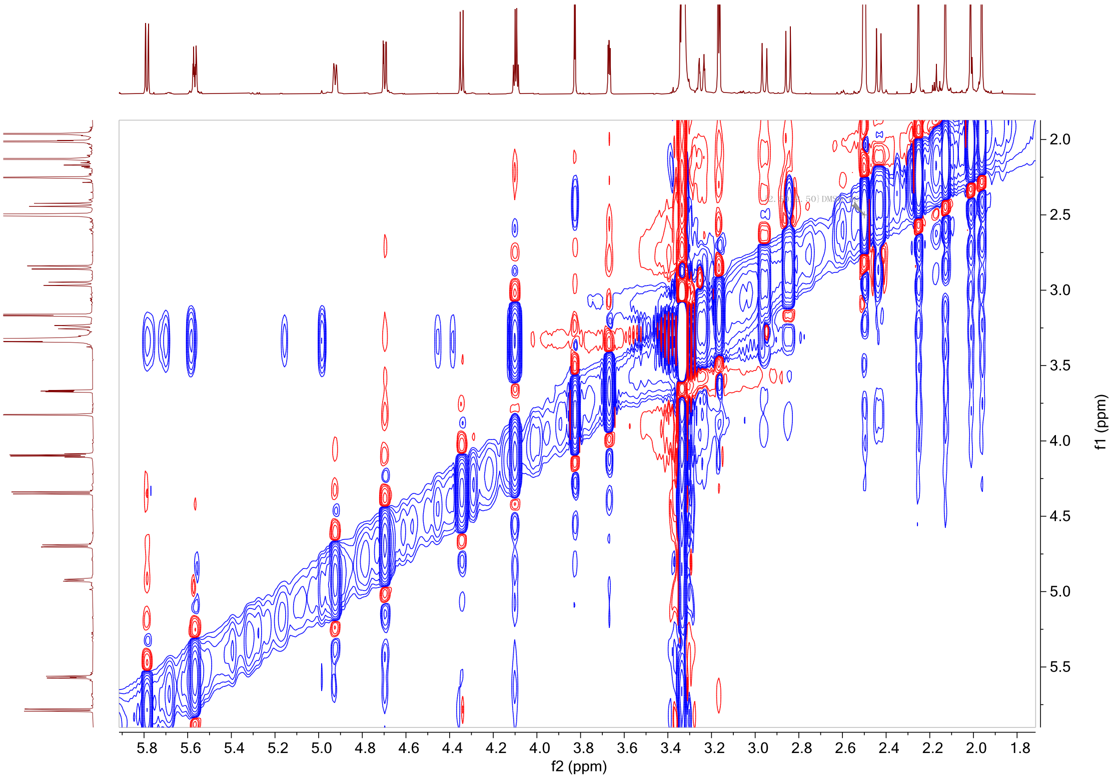

Figure S25 HR-ESIMS spectrum of compound **3**

Mass Spectrum SmartFormula Report

Analysis Info

Analysis Name	D:\Data\MS\data\202212\zhonglinfangQZLF-L-13_pos_54_01_14153.d	Acquisition Date	12/13/2022 5:28:59 PM
Method	LC_Direct Infusion_pos_70-500mz.m	Operator	SCSIO
Sample Name	zhonglinfangQZLF-L-13_pos	Instrument	maXis 255552.00029
Comment			

Acquisition Parameter

Source Type	ESI	Ion Polarity	Positive	Set Nebulizer	0.4 Bar
Focus	Active	Set Capillary	4500 V	Set Dry Heater	180 °C
Scan Begin	100 m/z	Set End Plate Offset	-500 V	Set Dry Gas	4.0 l/min
Scan End	1500 m/z	Set Charging Voltage	0 V	Set Divert Valve	Waste
		Set Corona	0 nA	Set APCI Heater	0 °C

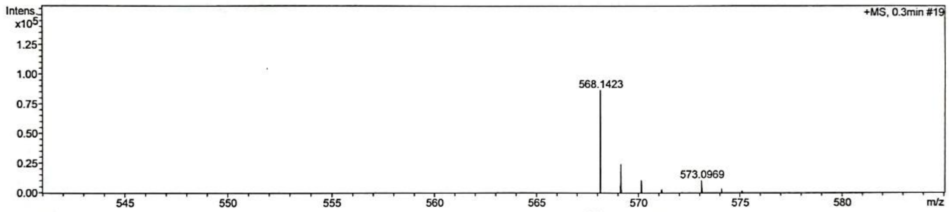

Meas. m/z	#	Ion Formula	Score	m/z	err [ppm]	err [mDa]	mSigma	rdb	e⁻ Conf	N-Rule
568.1423	1	C24H30N3O9S2	100.00	568.1418	-0.8	-0.5	12.5	11.5	even	ok
573.0969	1	C24H26N2NaO9S2	100.00	573.0972	-0.6	-0.3	24.4	12.5	even	ok
1118.2499	1	C48H56N5O18S4	100.00	1118.2498	-0.1	-0.1	19.3	23.5	even	ok
1123.2066	1	C48H52N4NaO18S4	100.00	1123.2052	1.3	1.4	29.6	24.5	even	ok

zhonglinfangQZLF-L-13_pos_54_01_14153.d
Bruker Compass DataAnalysis 4.1 printed: 12/13/2022 5:35:29 PM by: SCSIO Page 1 of 1

Figure S26 IR spectrum of compound **3**

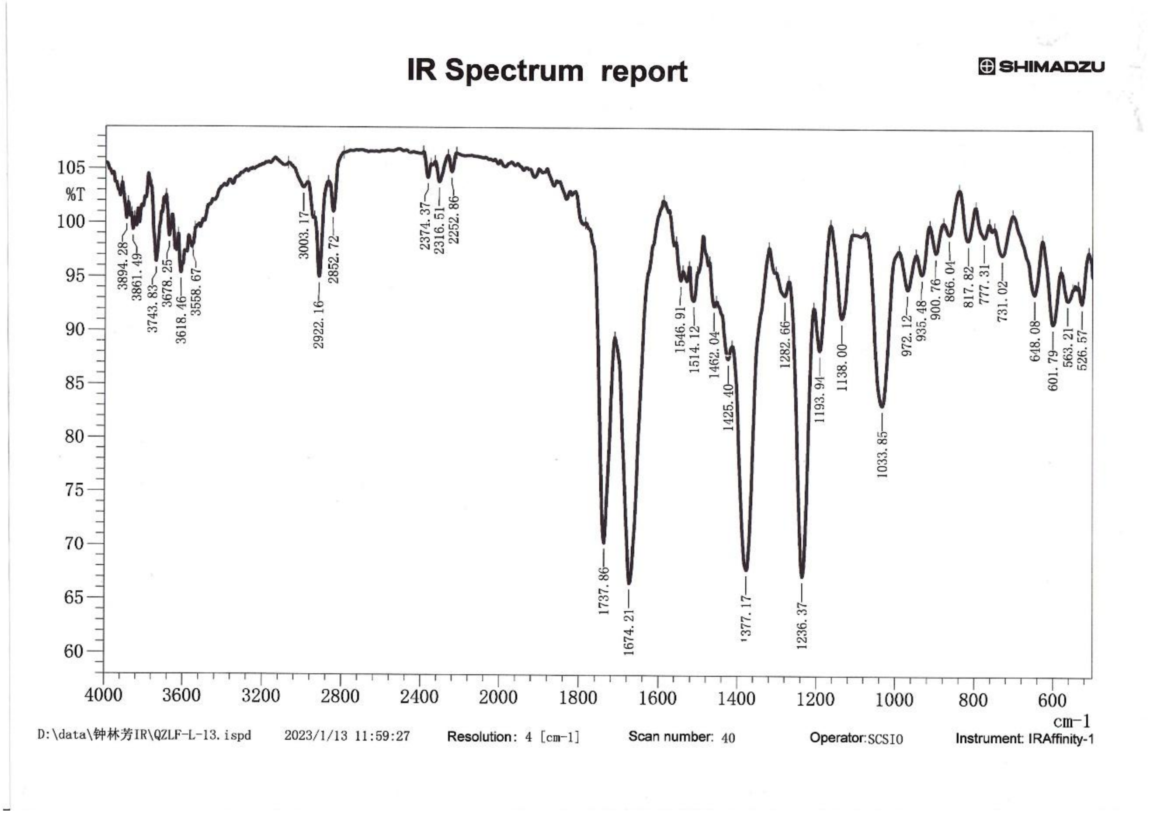

Figure S27 UV spectrum of compound **3**

光谱峰值检测报告 2023/01/12 13:23:45

数据集: QZLF-L-13 - RawData

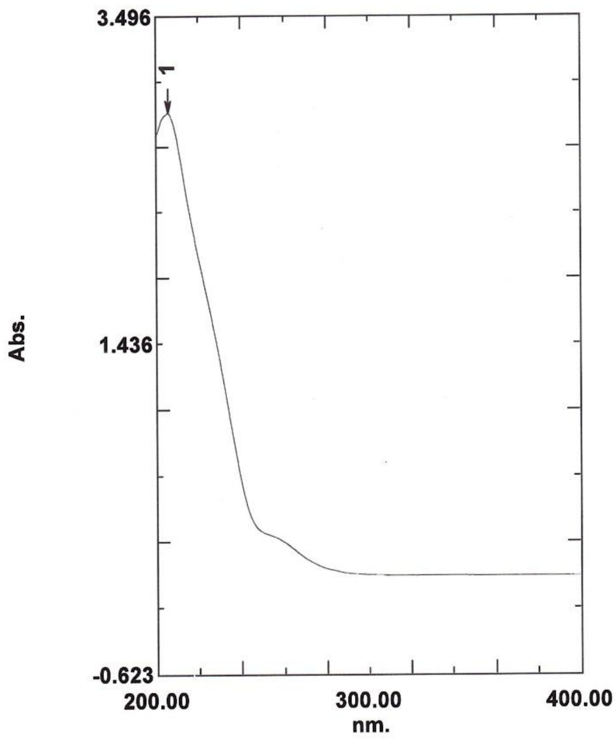

[测定属性]
波长范围(nm): 200.00 到 400.00
扫描速度: 中速
采样间隔: 0.2
自动采样间隔: 启用
扫描模式: 单个

[仪器属性]
仪器类型: UV-2600 系列
测定方式: 吸收值
狭缝宽: 2.0
积分时间: 0.1 秒.
光源转换波长: 323.0 nm
检测器单元: 直接
S/R 转换: 标准
阶梯校正: OFF

[附件属性]
附件: 无

[数据处理参数]
阈值: 0.0500000
点: 4
内插: 停用
平均: 停用

[样品准备属性]
重量:
体积:
稀释:
光程长:
附加信息:

No.	P/V	波长(nm)	Abs.	描述
1	⊕	205.80	2.887	

页 1 / 1

Figure S28 ^{1}H NMR spectrum of compound **4** in DMSO-d_6

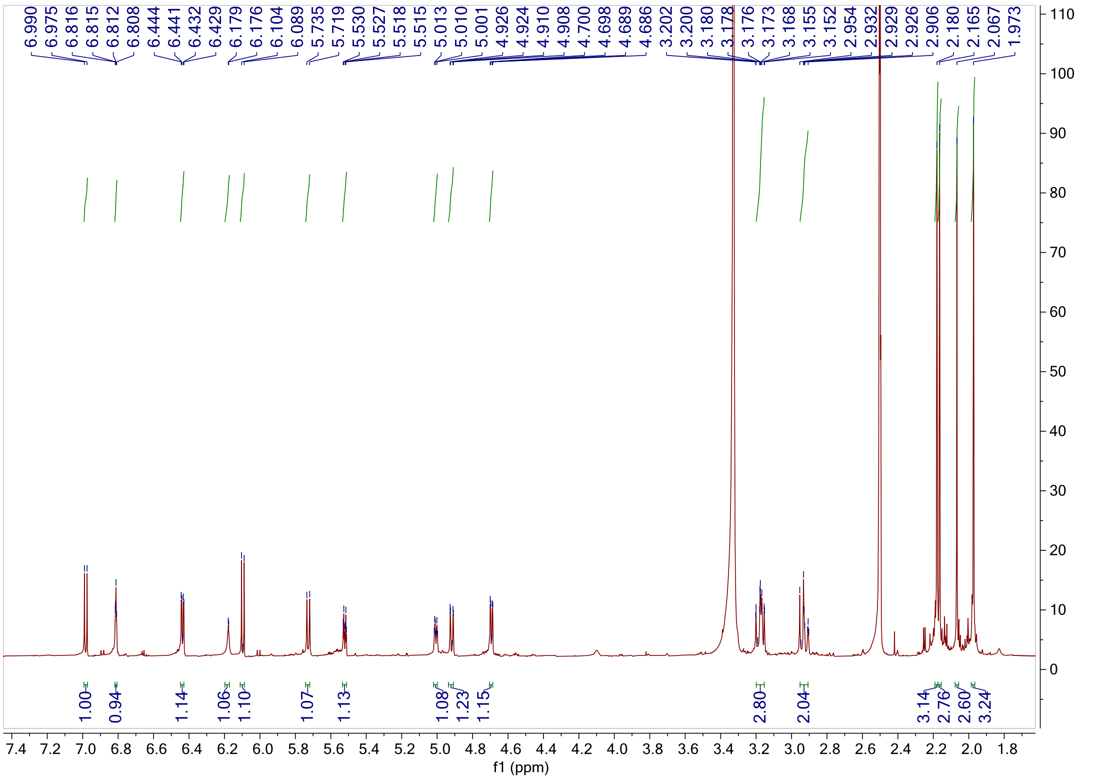

Figure S29 ^{13}C NMR spectrum of compound **4** in DMSO-d_6

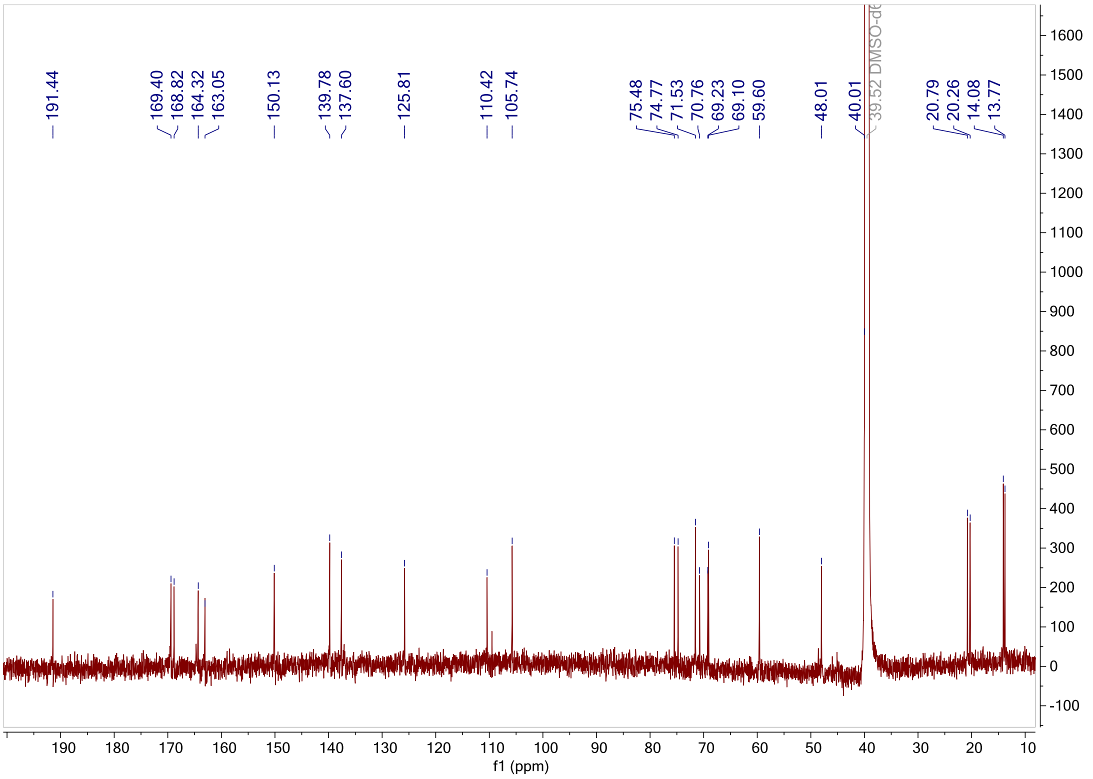

Figure S30 ^{1}H-^{1}H COSY spectrum of compound **4** in DMSO-d_6

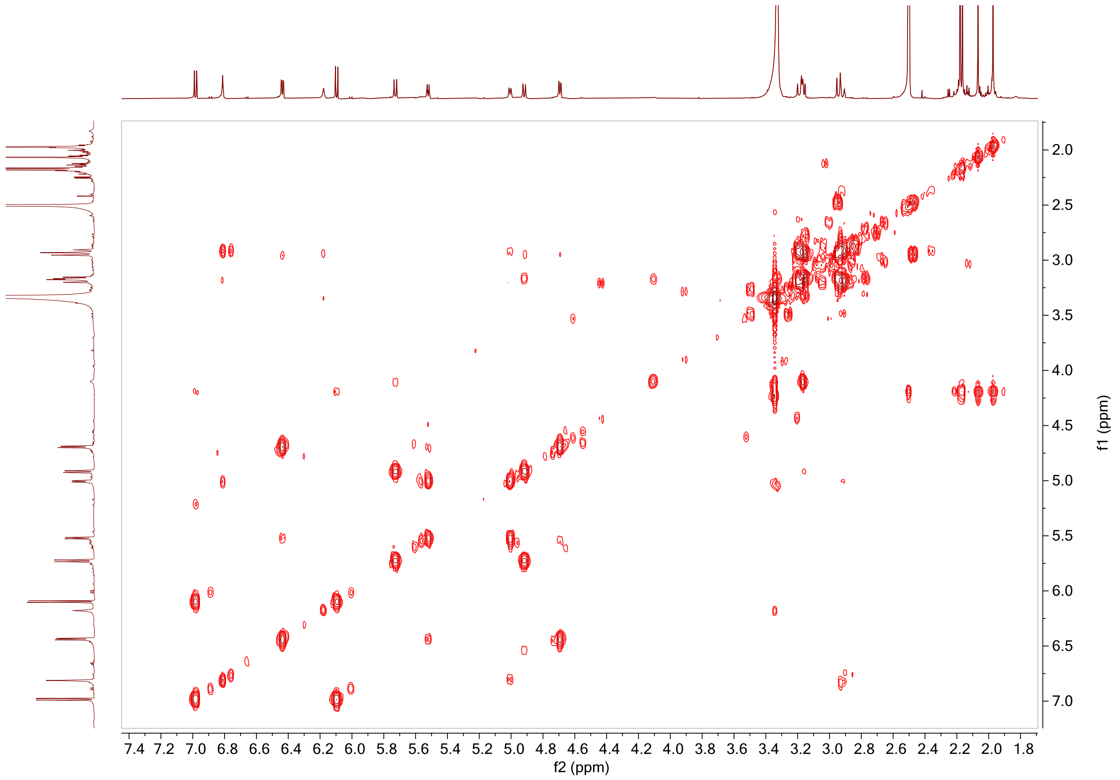

Figure S31 HSQC spectrum of compound **4** in DMSO-d_6

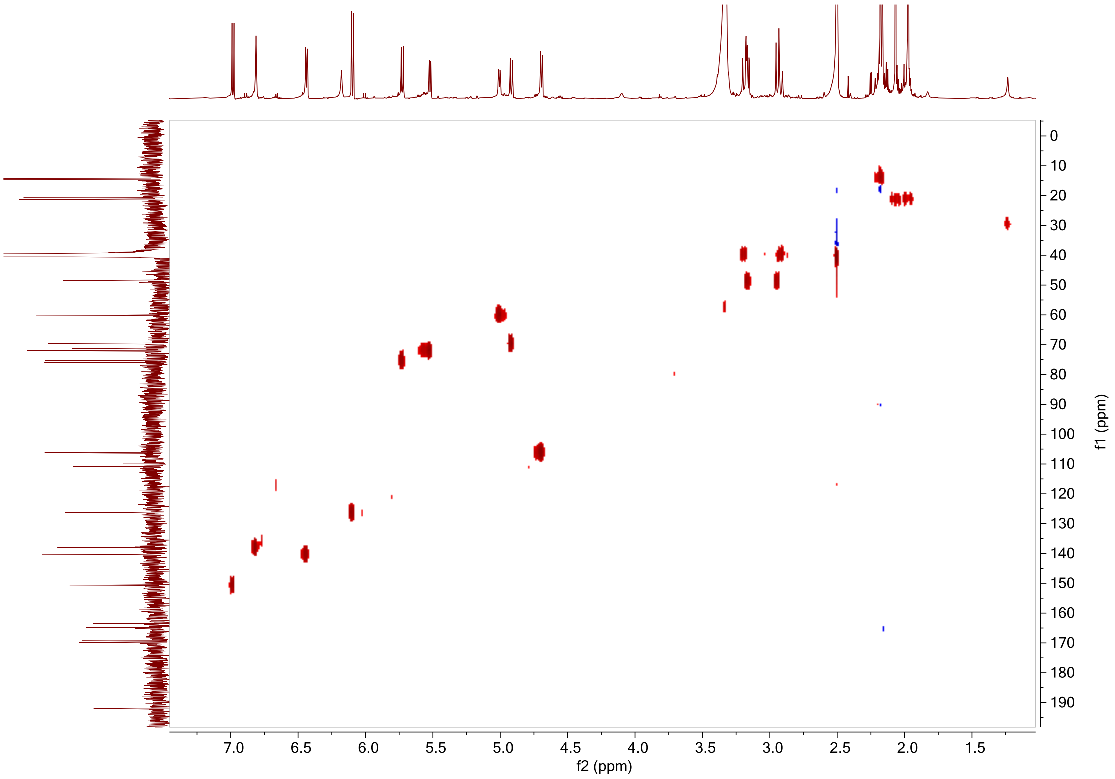

Figure S32 HMBC spectrum of compound **4** in DMSO-d_6

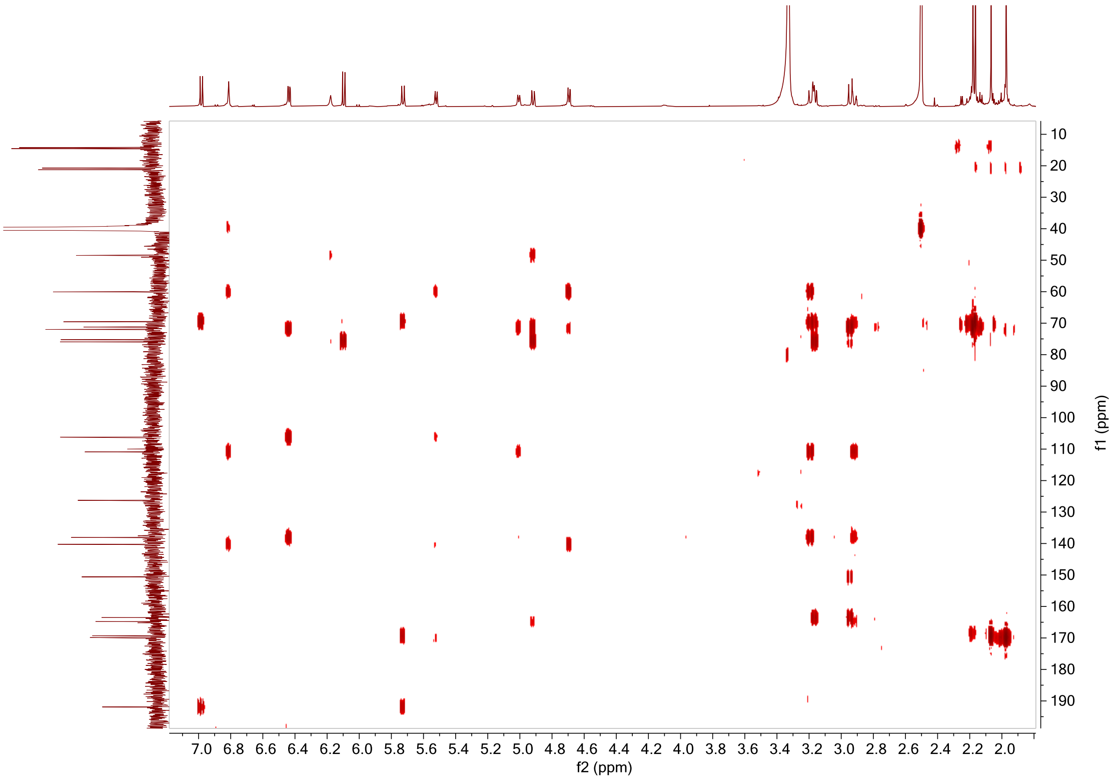

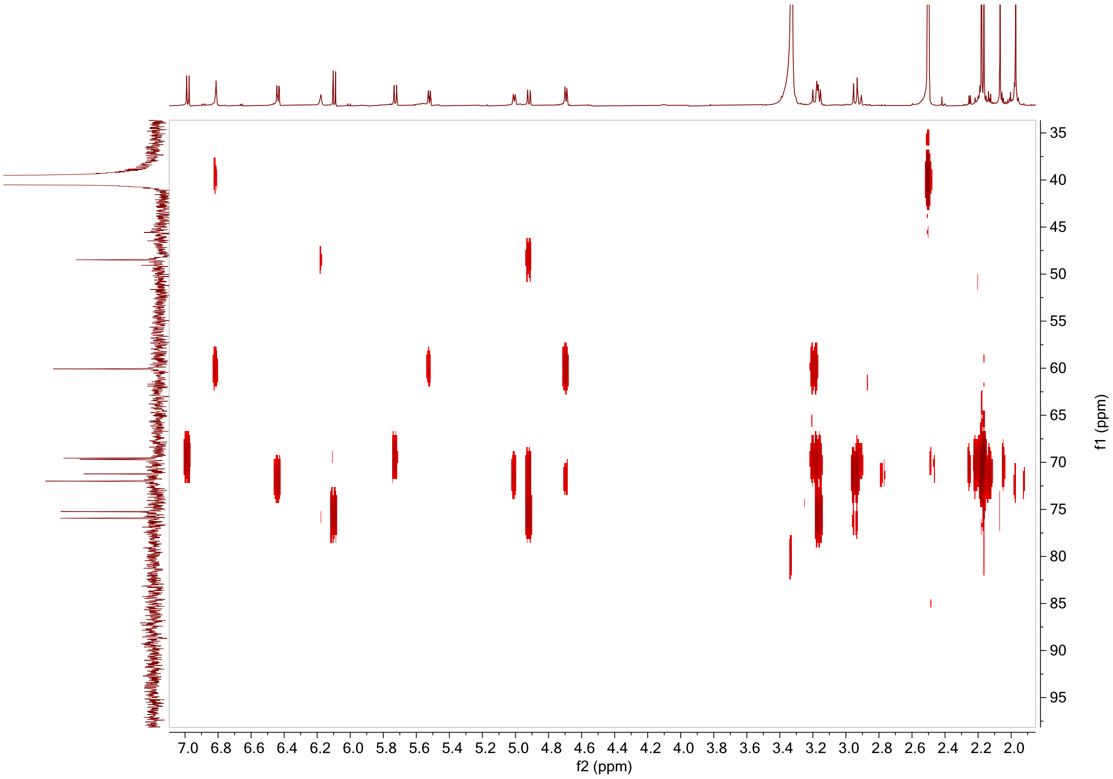

Figure S33 NOESY spectrum of compound **4** in DMSO-d_6

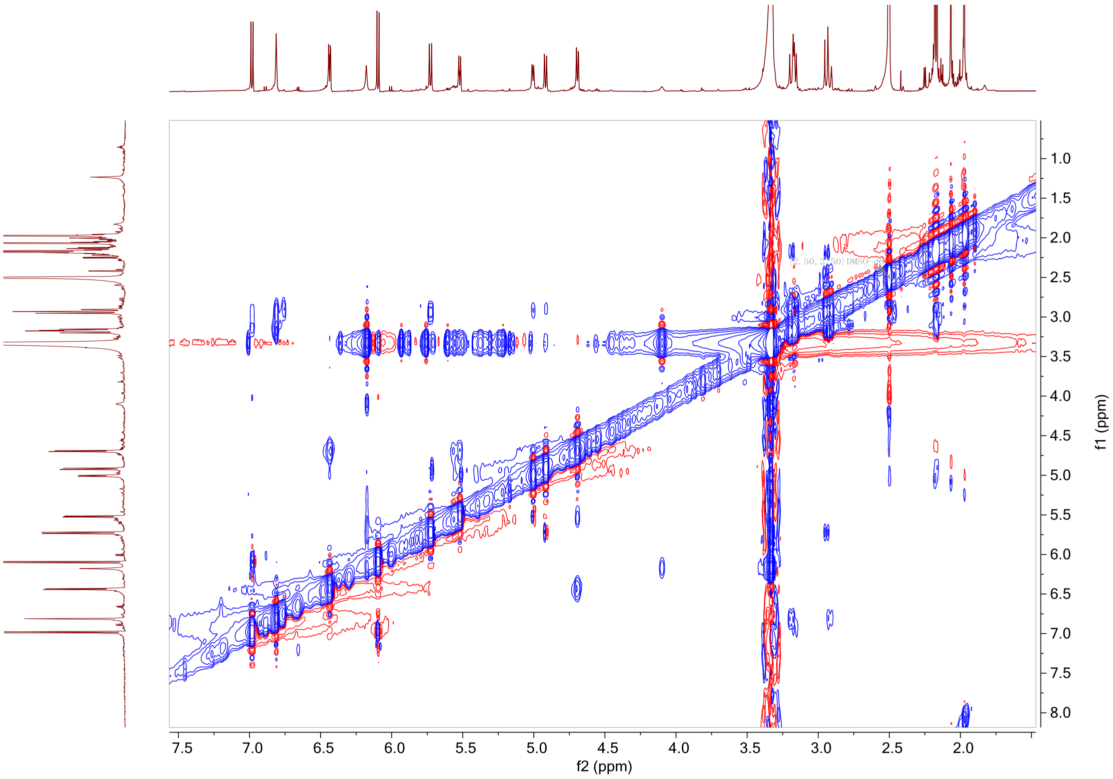

Figure S34 HR-ESIMS spectrum of compound **4**

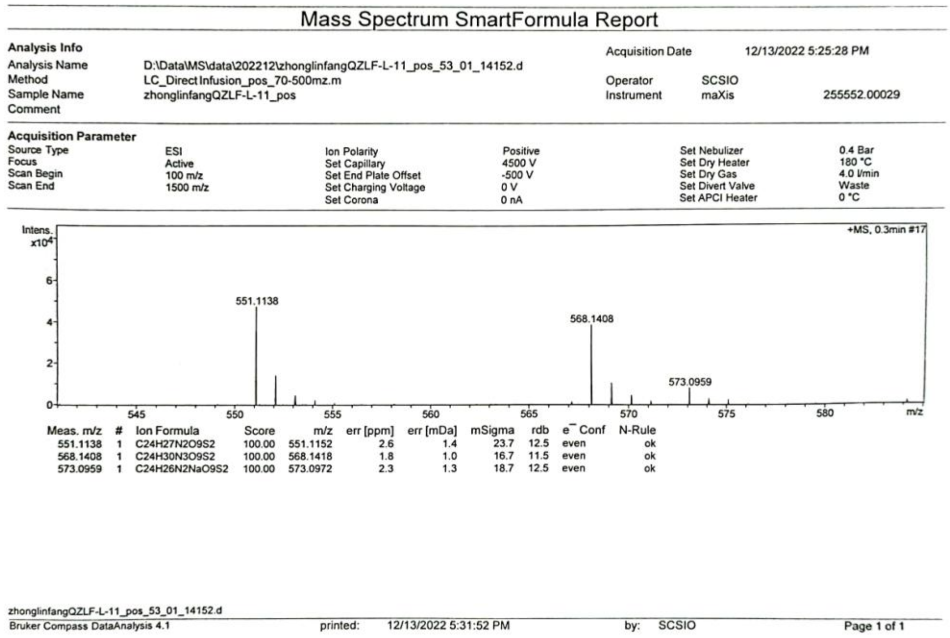

Mass Spectrum SmartFormula Report

Analysis Info

Analysis Name D:\Data\MS\data\202212\zhonglinfangQZLF-L-11_pos_53_01_14152.d
Method LC_Direct Infusion_pos_70-500mz.m
Sample Name zhonglinfangQZLF-L-11_pos
Comment

Acquisition Date 12/13/2022 5:25:28 PM
Operator SCSIO
Instrument maXis 255552.00029

Acquisition Parameter

Source Type	ESI	Ion Polarity	Positive	Set Nebulizer	0.4 Bar
Focus	Active	Set Capillary	4500 V	Set Dry Heater	180 °C
Scan Begin	100 m/z	Set End Plate Offset	-500 V	Set Dry Gas	4.0 l/min
Scan End	1500 m/z	Set Charging Voltage	0 V	Set Divert Valve	Waste
		Set Corona	0 nA	Set APCI Heater	0 °C

Meas. m/z	#	Ion Formula	Score	m/z	err [ppm]	err [mDa]	mSigma	rdb	e⁻ Conf	N-Rule
551.1138	1	$C_{24}H_{27}N_2O_9S_2$	100.00	551.1152	2.6	1.4	23.7	12.5	even	ok
568.1408	1	$C_{24}H_{30}N_3O_9S_2$	100.00	568.1418	1.8	1.0	16.7	11.5	even	ok
573.0959	1	$C_{24}H_{26}N_2NaO_9S_2$	100.00	573.0972	2.3	1.3	18.7	12.5	even	ok

zhonglinfangQZLF-L-11_pos_53_01_14152.d
Bruker Compass DataAnalysis 4.1 printed: 12/13/2022 5:31:52 PM by: SCSIO Page 1 of 1

Figure S35 IR spectrum of compound **4**

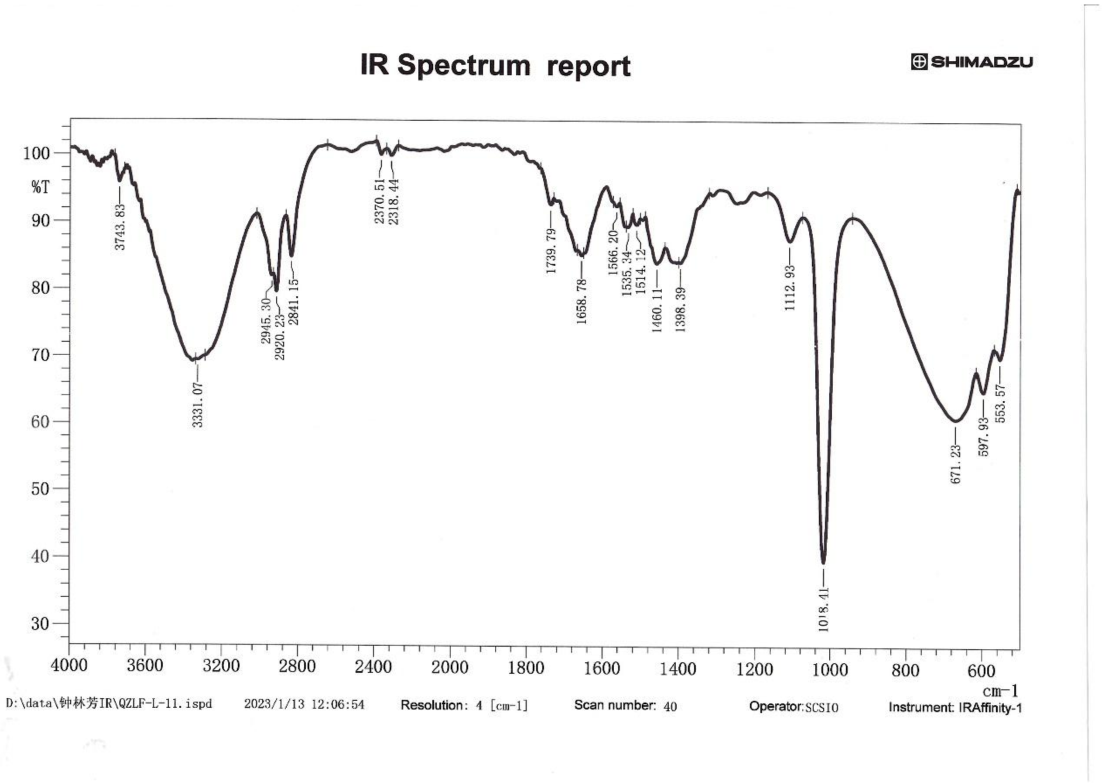

Figure S36 UV spectrum of compound **4**

光谱峰值检测报告

2023/01/12 12:55:32

数据集: QZLF-L-11 - RawData

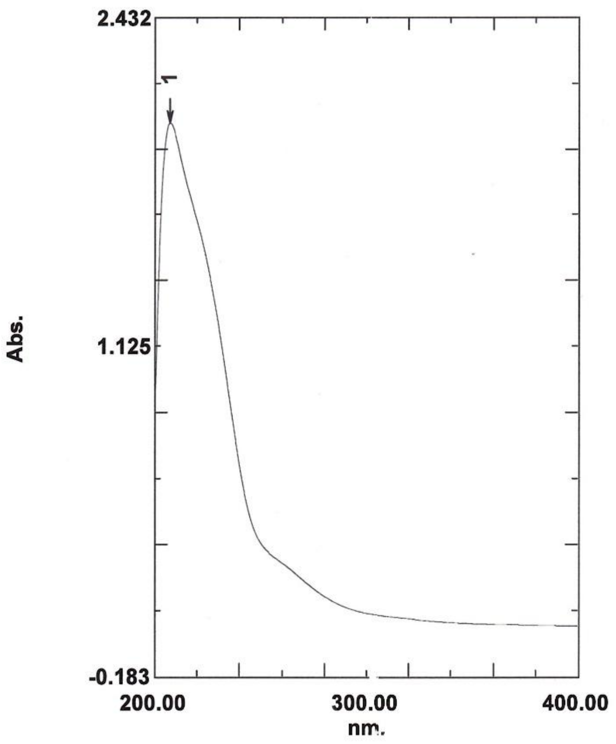

[测定属性]
波长范围(nm): 200.00 到 400.00
扫描速度: 中速
采样间隔: 0.2
自动采样间隔: 启用
扫描模式: 单个

[仪器属性]
仪器类型: UV-2600 系列
测定方式: 吸收值
狭缝宽: 2.0
积分时间: 0.1 秒.
光源转换波长: 323.0 nm
检测器单元: 直接
S/R 转换: 标准
阶梯校正: OFF

[附件属性]
附件: 无

[数据处理参数]
阈值: 0.0500000
点: 4
内插: 停用
平均: 停用

[样品准备属性]
重量:
体积:
稀释:
光程长:
附加信息:

No.	P/V	波长(nm)	Abs.	描述
1	⬆	207.20	2.013	

页 1 / 1

Figure S37 ^{1}H NMR spectrum of compound **5** in DMSO-d_6

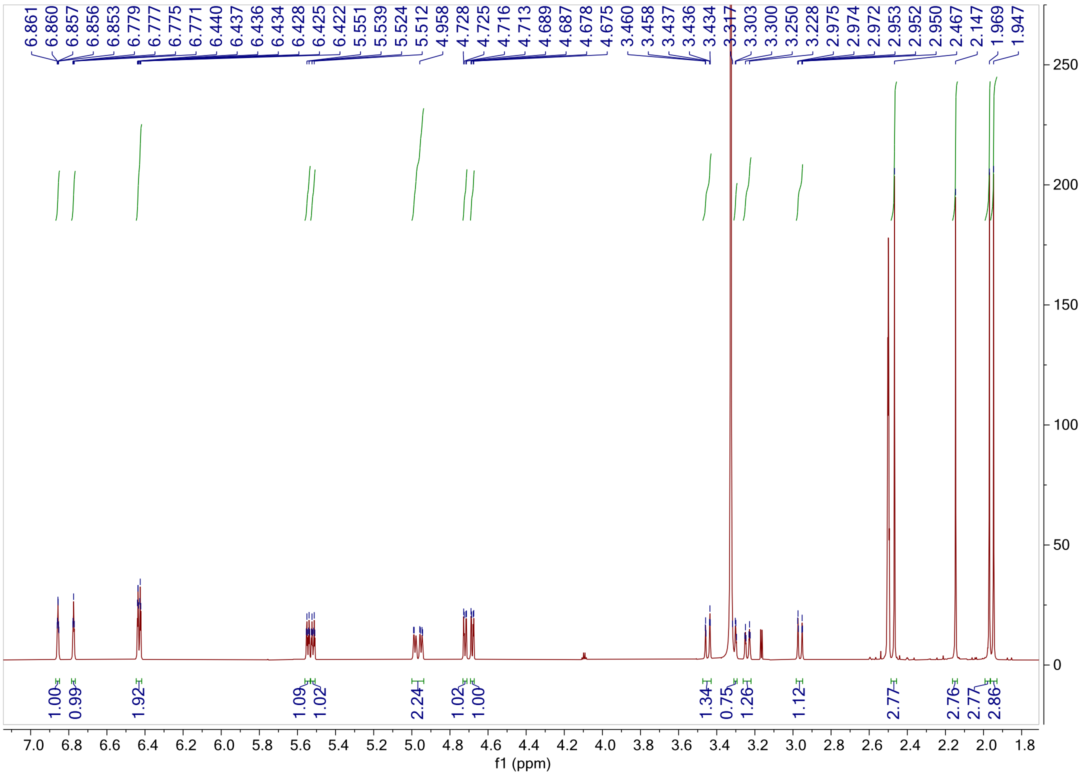

Figure S38 ^{13}C NMR spectrum of compound **5** in DMSO-d_6

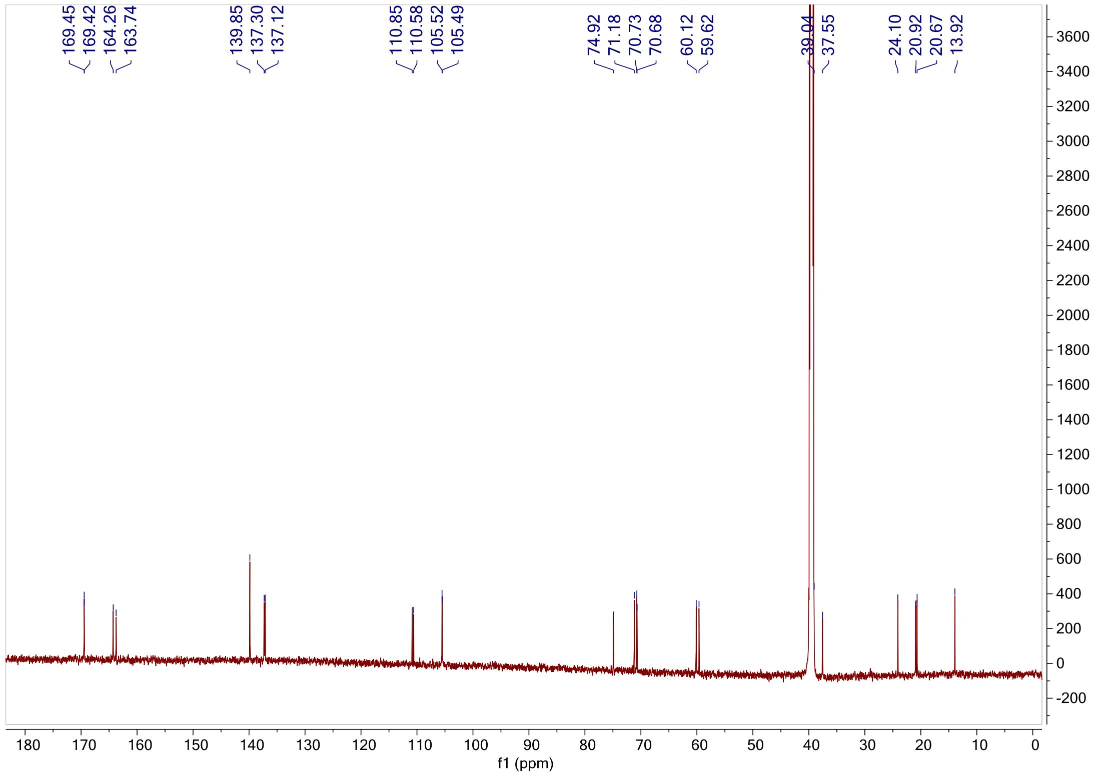

Figure S39 ^{1}H-^{1}H COSY spectrum of compound **5** in DMSO-d_6

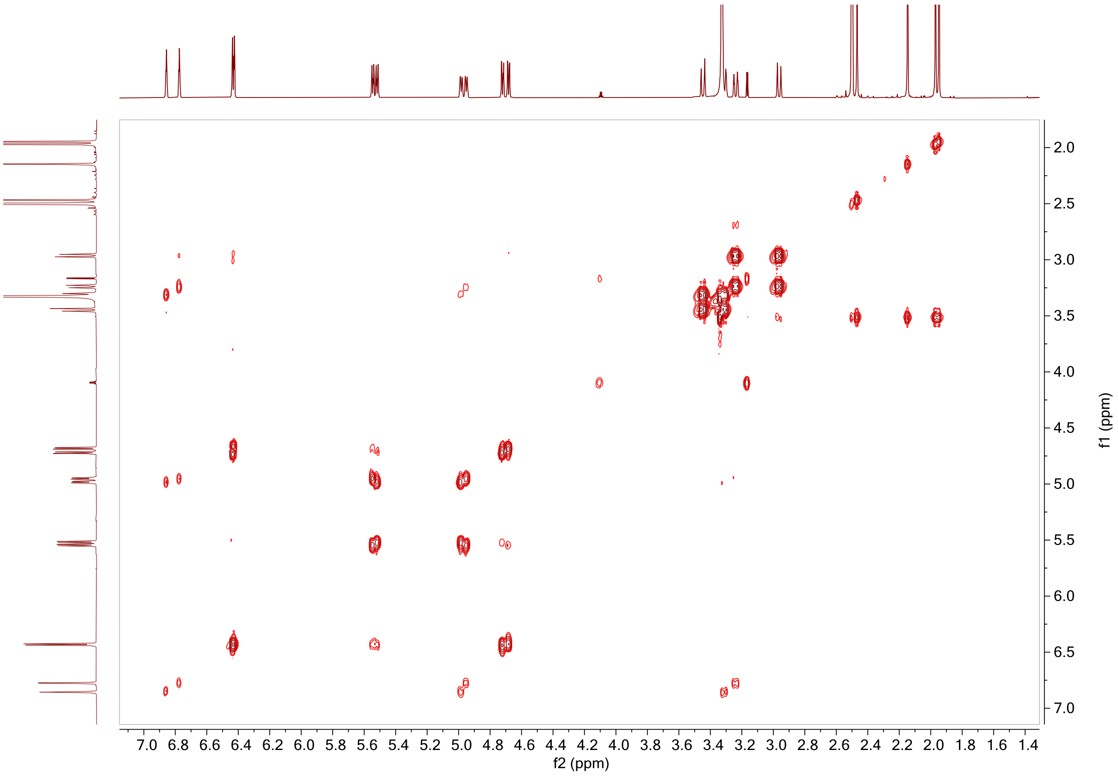

Figure S40 HSQC spectrum of compound **5** in DMSO-d_6

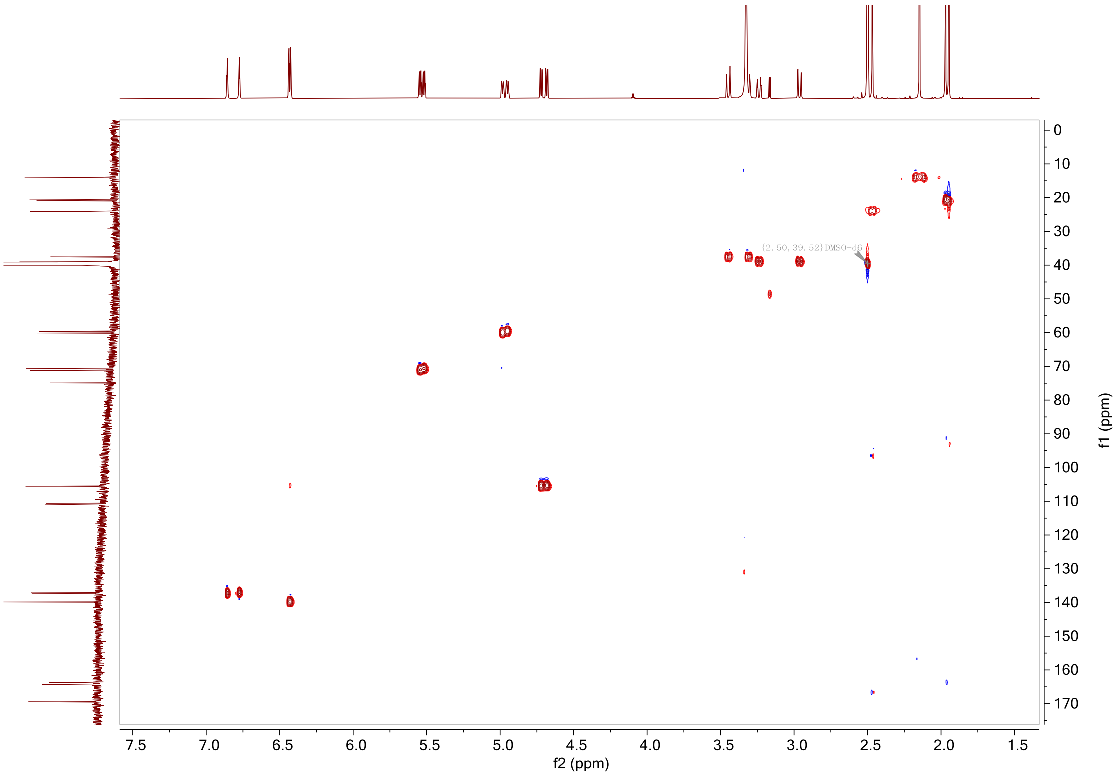

Figure S41 HMBC spectrum of compound **5** in DMSO-d_6

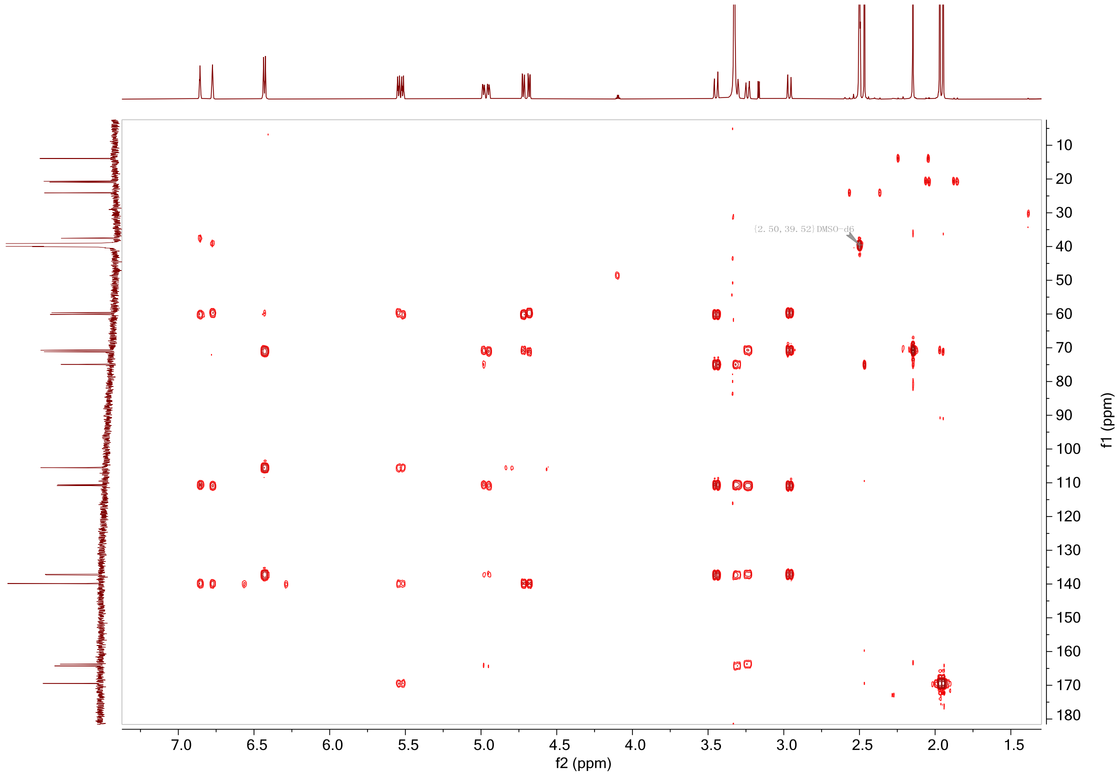

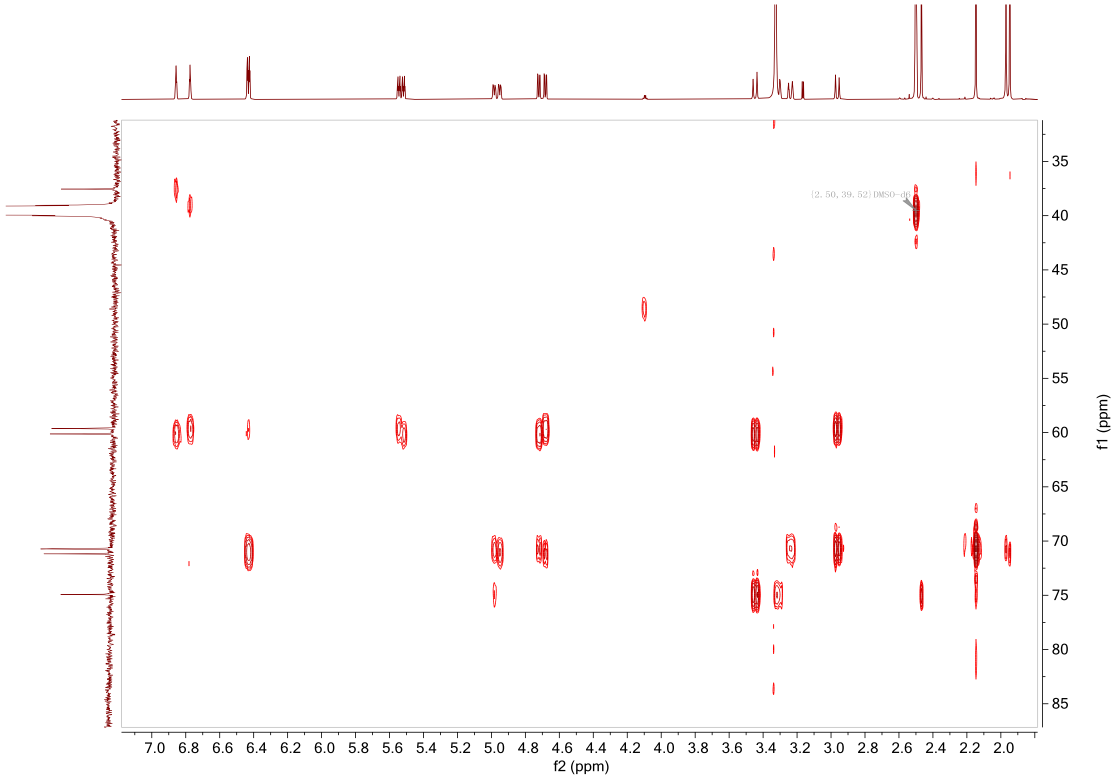

Figure S42 NOESY spectrum of compound **5** in DMSO-d_6

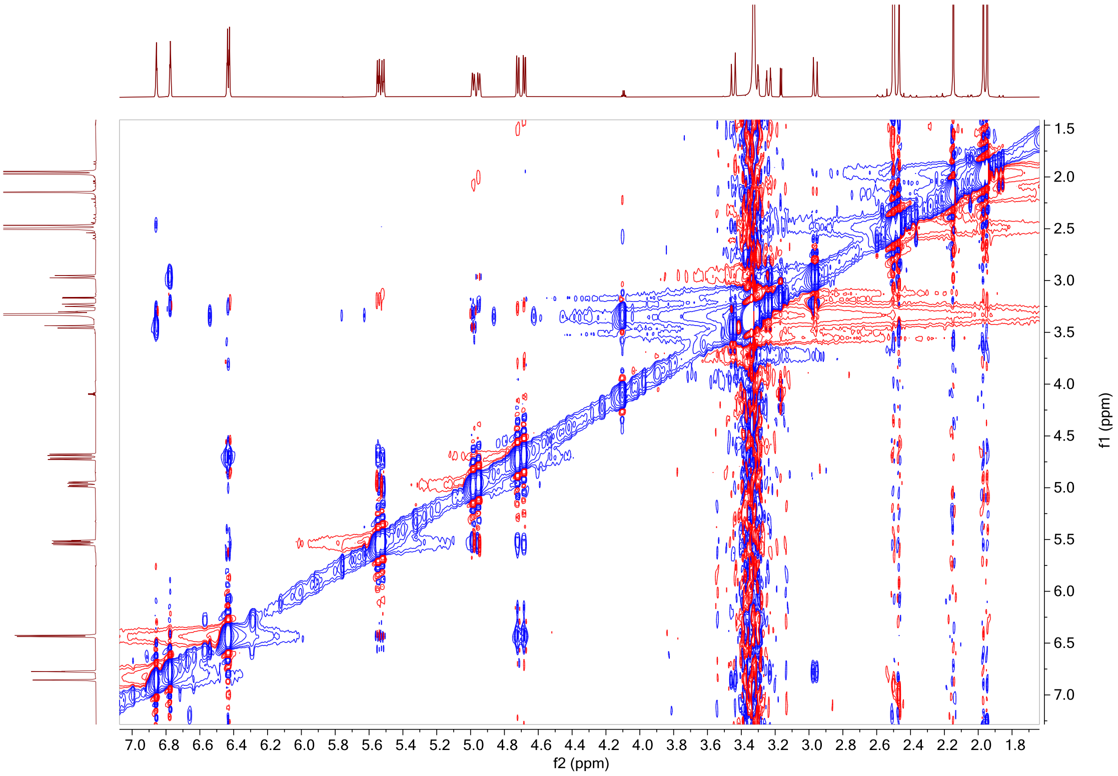

Figure S43 HR-ESIMS spectrum of compound **5**

Mass Spectrum SmartFormula Report

Analysis Info

Analysis Name	D:\Data\MS\data\202210\zhonglinfang_QZLF-L-8_pos_75_01_13864.d	Acquisition Date	10/13/2022 9:13:59 AM
Method	LC_Direct Infusion_pos_70-500mz.m	Operator	SCSIO
Sample Name	zhonglinfang_QZLF-L-8_pos	Instrument	maXis 255552.00029
Comment			

Acquisition Parameter

Source Type	ESI	Ion Polarity	Positive	Set Nebulizer	0.4 Bar
Focus	Active	Set Capillary	4500 V	Set Dry Heater	180 °C
Scan Begin	100 m/z	Set End Plate Offset	-500 V	Set Dry Gas	4.0 l/min
Scan End	1500 m/z	Set Charging Voltage	0 V	Set Divert Valve	Waste
		Set Corona	0 nA	Set APCI Heater	0 °C

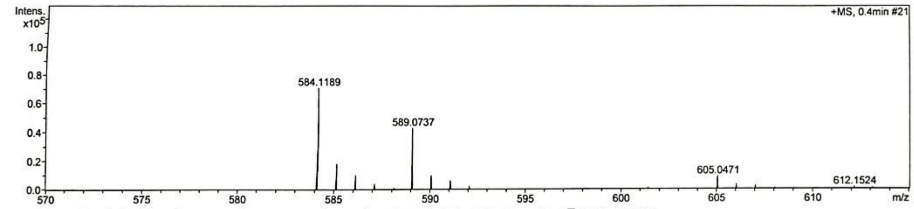

Meas. m/z	#	Ion Formula	Score	m/z	err [ppm]	err [mDa]	mSigma	rdb	e⁻ Conf	N-Rule
567.0930	1	C24H27N2O8S3	100.00	567.0924	-1.0	-0.6	143.6	12.5	even	ok
584.1189	1	C24H30N3O8S3	100.00	584.1190	0.0	0.0	31.5	11.5	even	ok
589.0737	1	C24H26N2NaO8S3	100.00	589.0743	-1.0	-0.6	38.6	12.5	even	ok
1150.2025	1	C48H56N5O16S6	100.00	1150.2041	1.3	1.5	21.9	23.5	even	ok
1155.1610	1	C48H52N4NaO16S6	100.00	1155.1595	-1.3	-1.5	51.7	24.5	even	ok

zhonglinfang_QZLF-L-8_pos_75_01_13864.d

Bruker Compass DataAnalysis 4.1 printed: 10/13/2022 9:20:56 AM by: SCSIO Page 1 of 1

Figure S44 IR spectrum of compound **5**

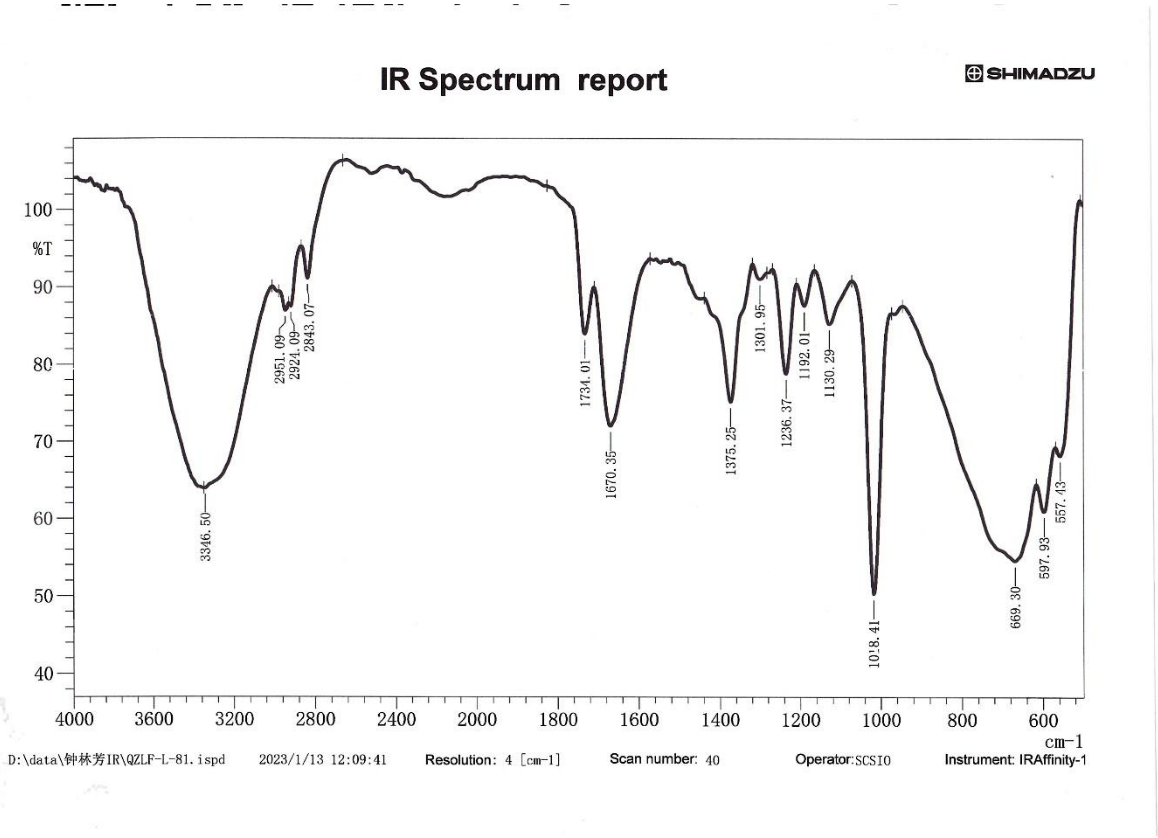

Figure S45 UV spectrum of compound **5**

光谱峰值检测报告

2023/01/12 12:42:57

数据集: QZLF-L-8 - RawData

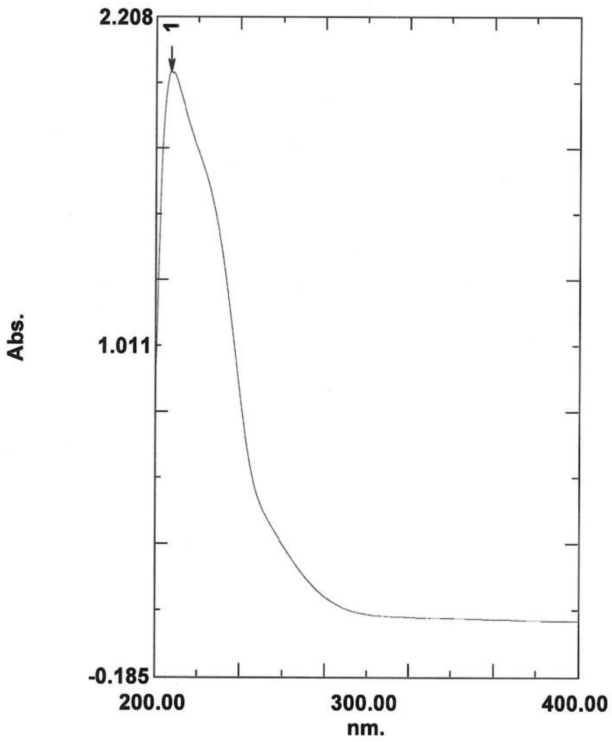

[测定属性]
波长范围(nm): 200.00 到 400.00
扫描速度: 中速
采样间隔: 0.2
自动采样间隔: 启用
扫描模式: 单个

[仪器属性]
仪器类型: UV-2600 系列
测定方式: 吸收值
狭缝宽: 2.0
积分时间: 0.1 秒.
光源转换波长: 323.0 nm
检测器单元: 直接
S/R 转换: 标准
阶梯校正: OFF

[附件属性]
附件: 无

[数据处理参数]
阈值: 0.0500000
点: 4
内插: 停用
平均: 停用

[样品准备属性]
重量:
体积:
稀释:
光程长:
附加信息:

No.	P/V	波长(nm)	Abs.	描述
1	⬆	206.80	2.009	

页 1 / 1

Figure S46 ^{1}H NMR spectrum of compound **6** in DMSO-d_6

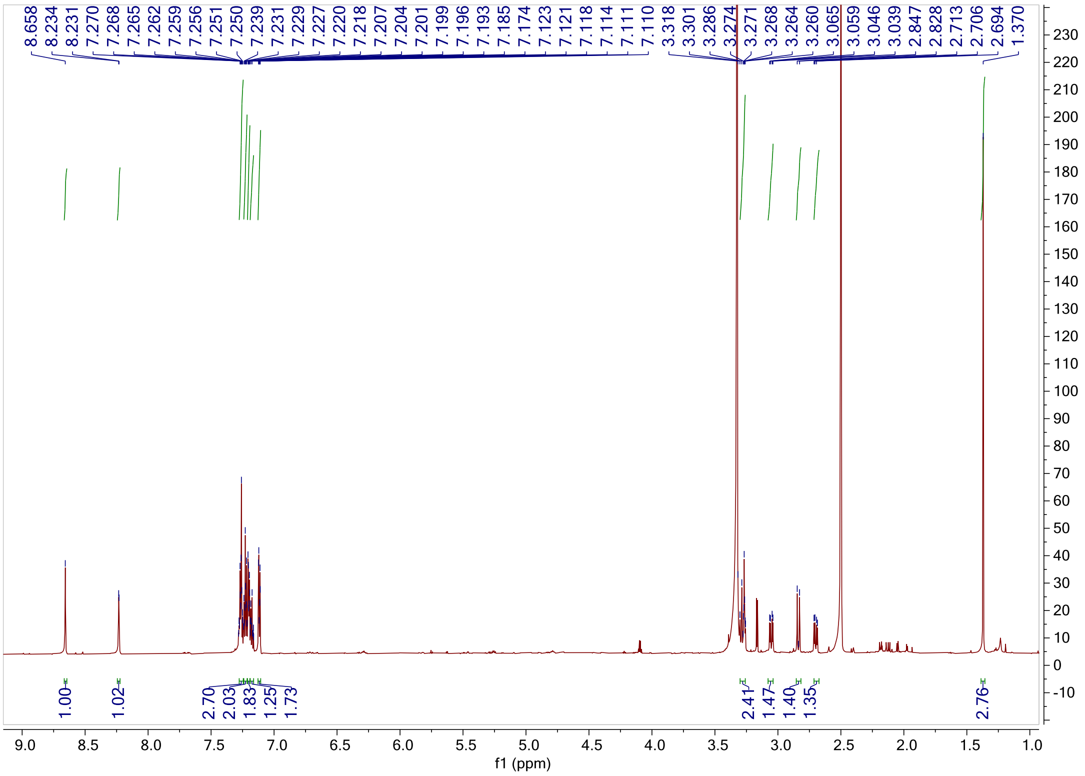

Figure S47 ^{13}C NMR spectrum of compound **6** in DMSO-d_6

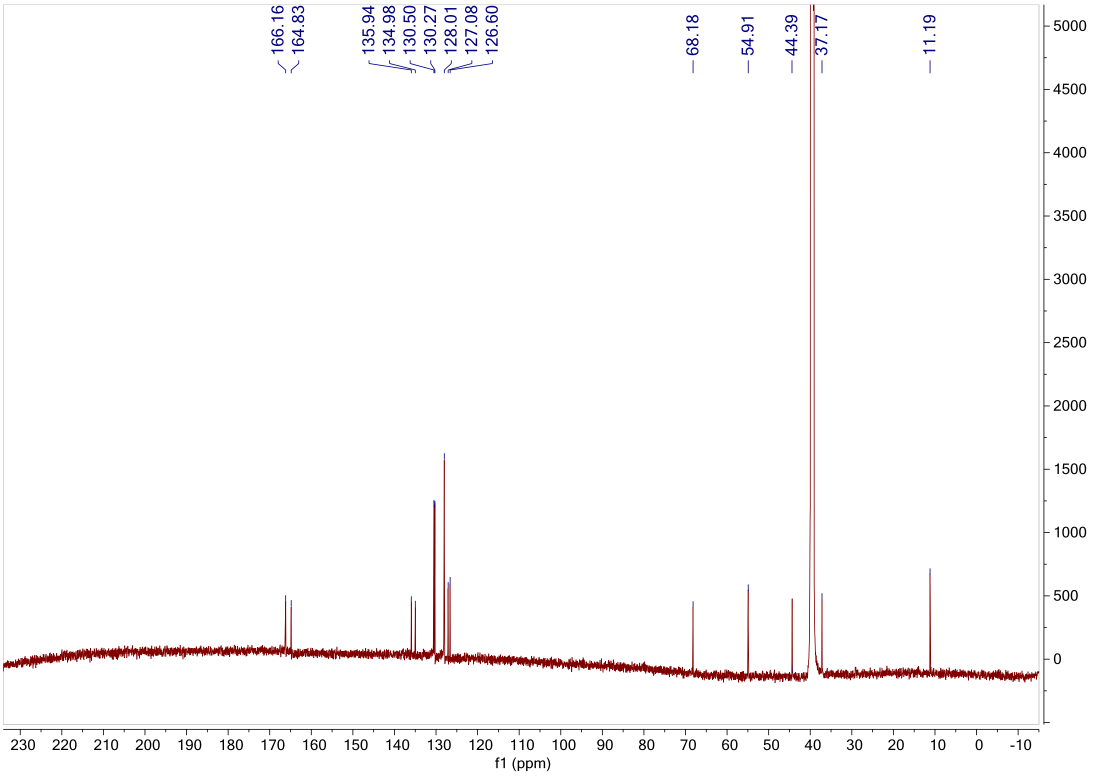

Figure S48 ^{1}H-^{1}H COSYspectrum of compound **6** in DMSO-d_6

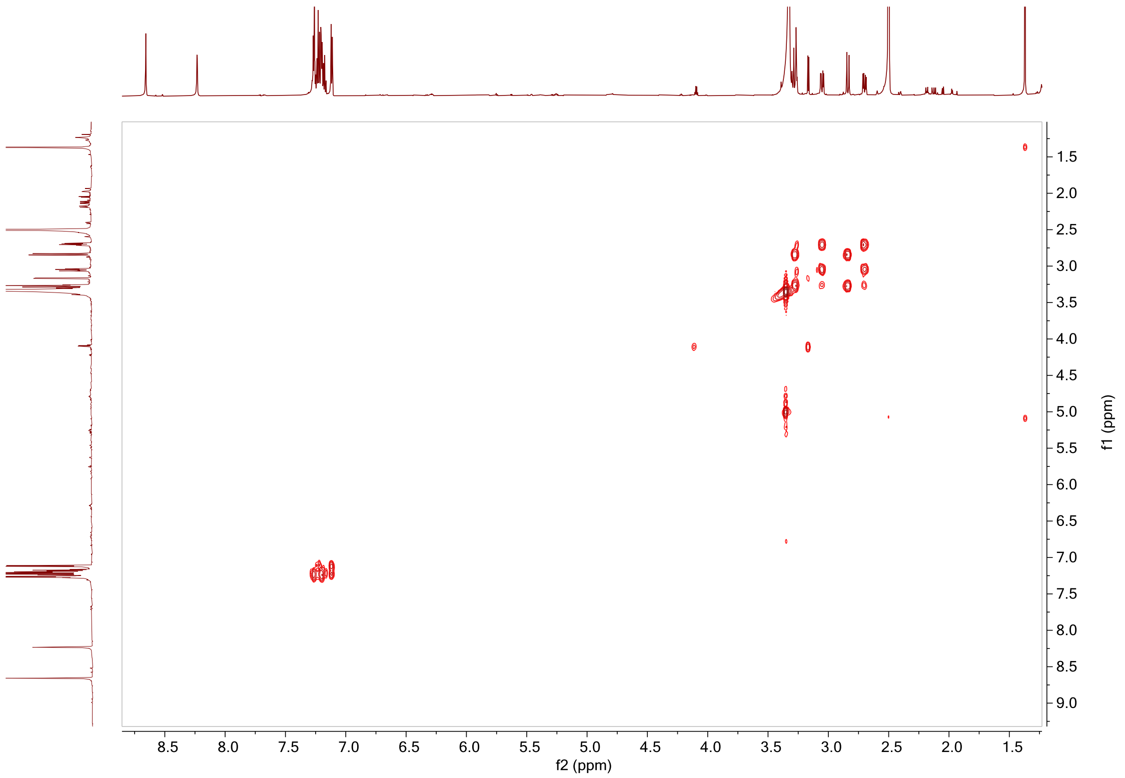

Figure S49 HSQC spectrum of compound **6** in DMSO-d_6

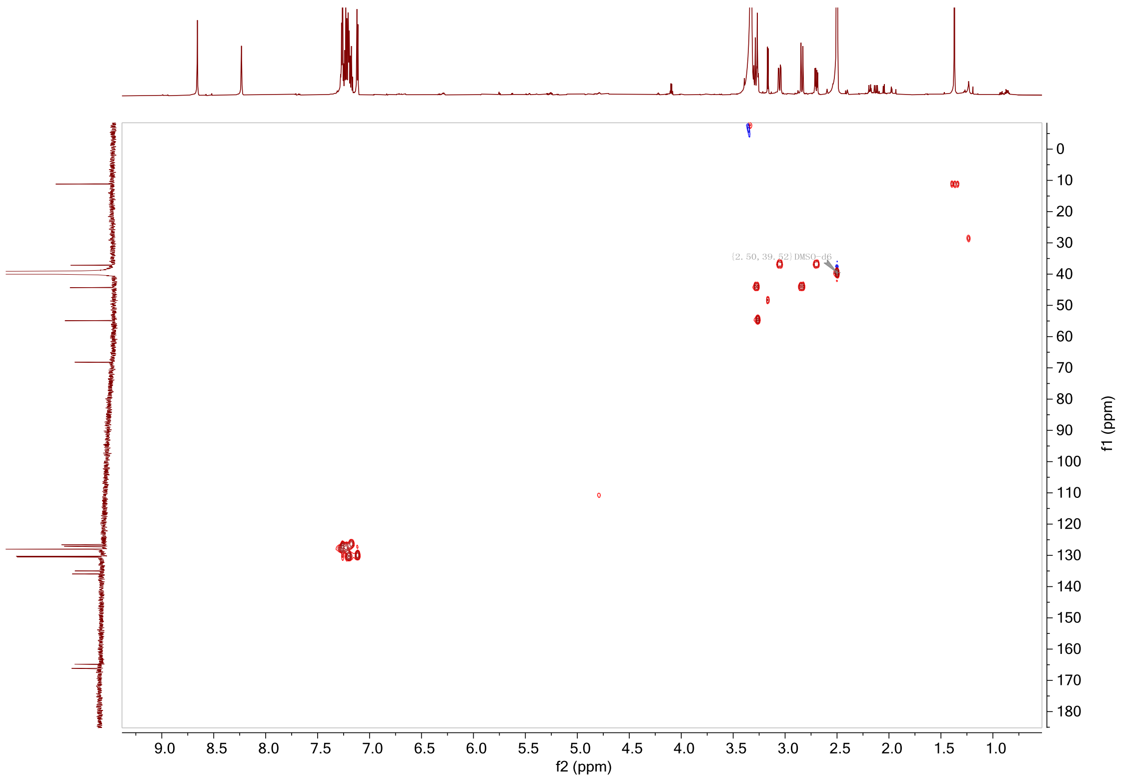

Figure S50 HMBC spectrum of compound **6** in DMSO-d_6

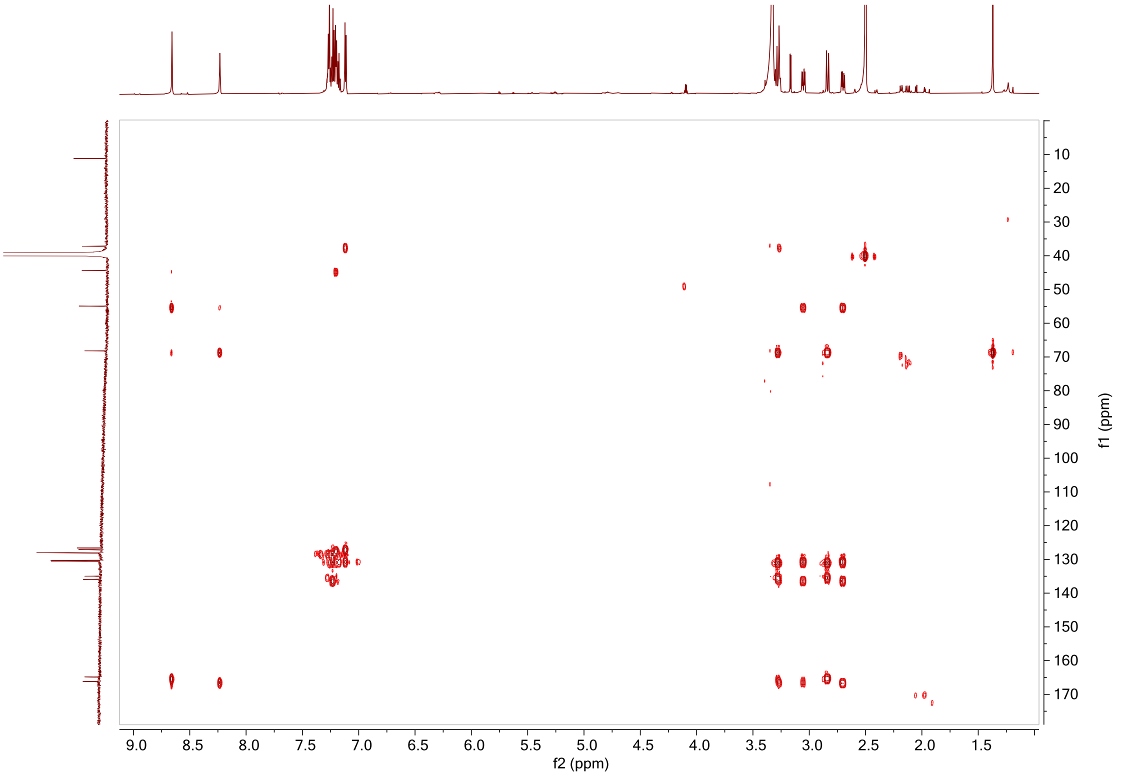

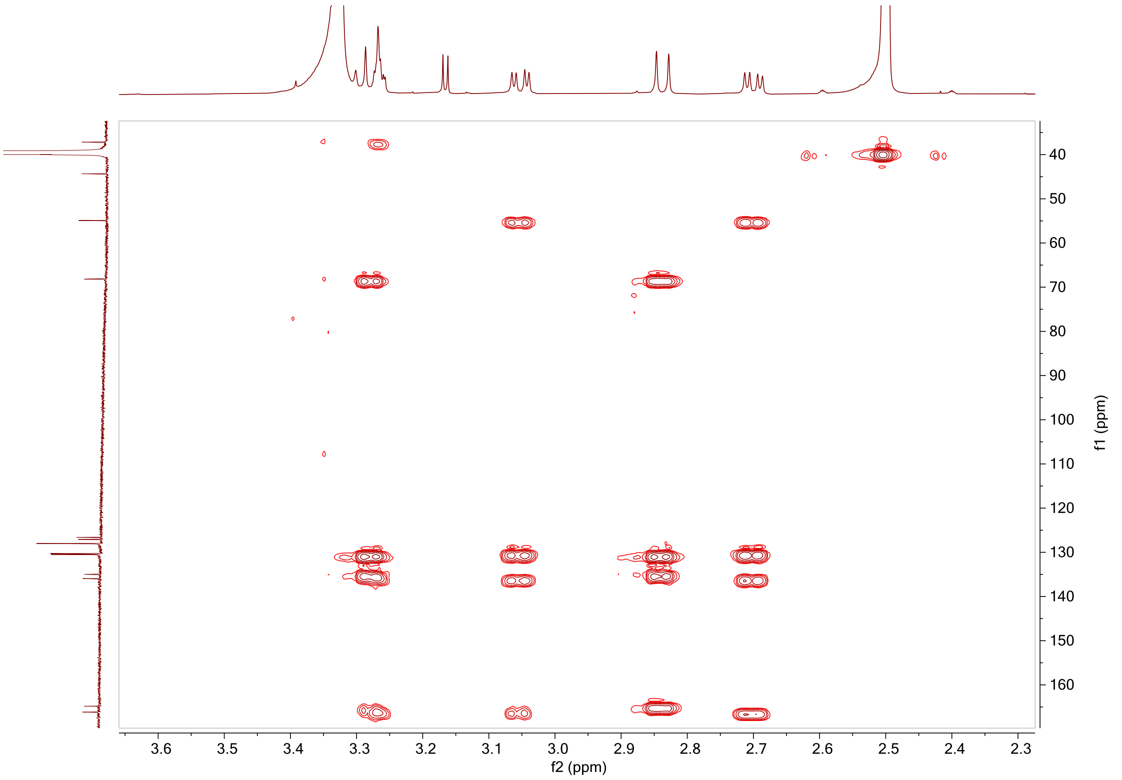

Figure S51 NOESY spectrum of compound **6** in DMSO-d_6

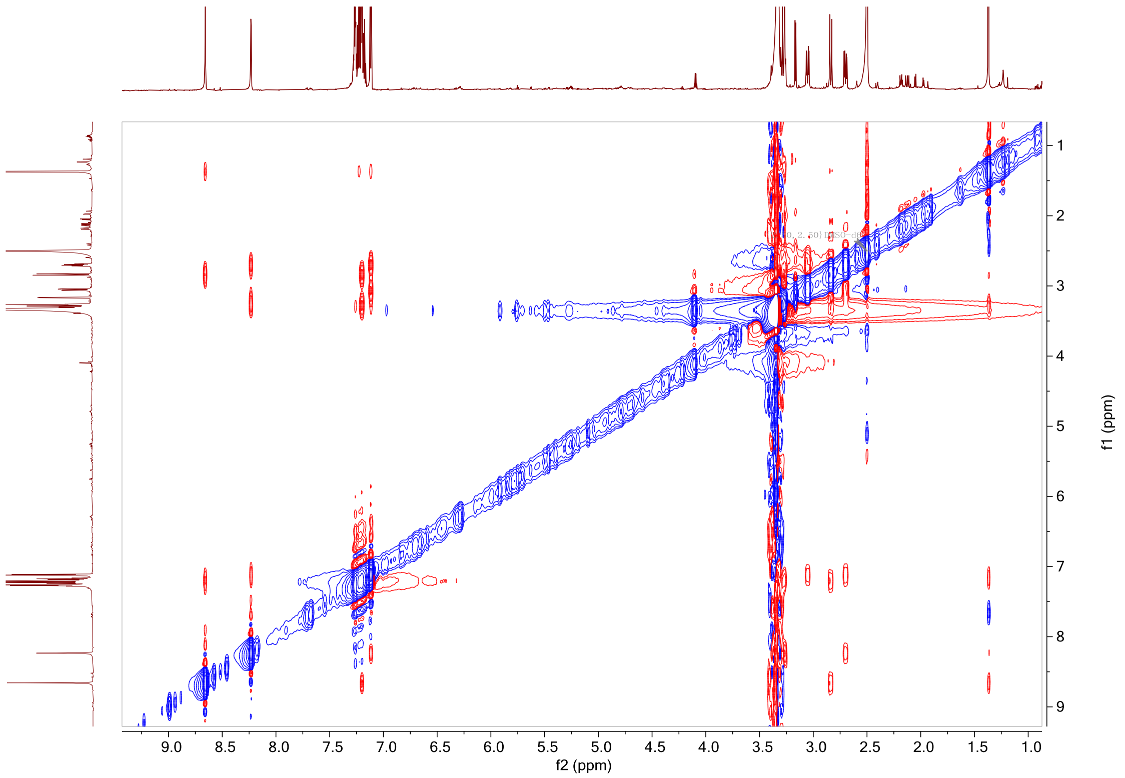

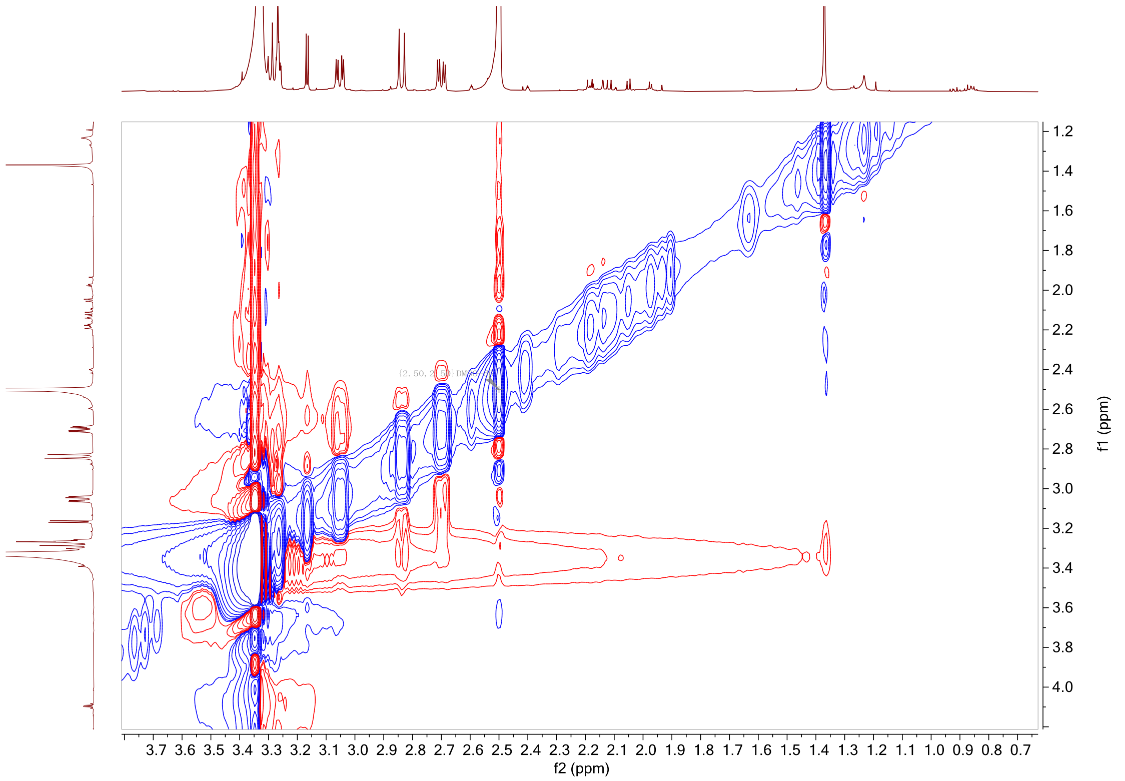

Figure S52 HR-ESIMS spectrum of compound **6**

Mass Spectrum SmartFormula Report

Analysis Info

Analysis Name	D:\Data\MS\data\202212\zhonglinfangQZLF-L-17_pos_56_01_14151.d	Acquisition Date	12/13/2022 5:15:21 PM
Method	LC_Direct Infusion_pos_70-500mz.m	Operator	SCSIO
Sample Name	zhonglinfangQZLF-L-17_pos	Instrument	maXis 255552.00029
Comment			

Acquisition Parameter

Source Type	ESI	Ion Polarity	Positive	Set Nebulizer	0.4 Bar
Focus	Active	Set Capillary	4500 V	Set Dry Heater	180 °C
Scan Begin	100 m/z	Set End Plate Offset	-500 V	Set Dry Gas	4.0 l/min
Scan End	1500 m/z	Set Charging Voltage	0 V	Set Divert Valve	Waste
		Set Corona	0 nA	Set APCI Heater	0 °C

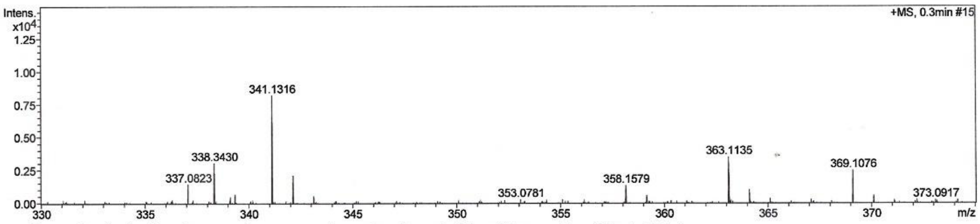

Meas. m/z	#	Ion Formula	Score	m/z	err [ppm]	err [mDa]	mSigma	rdb	e⁻ Conf	N-Rule
341.1316	1	C19H21N2O2S	100.00	341.1318	-0.7	-0.3	17.6	10.5	even	ok
363.1135	1	C19H20N2NaO2S	100.00	363.1138	0.8	0.3	41.3	10.5	even	ok
681.2558	1	C38H41N4O4S2	100.00	681.2564	0.8	0.6	63.2	20.5	even	ok
703.2378	1	C38H40N4NaO4S2	100.00	703.2383	0.7	0.5	46.2	20.5	even	ok

zhonglinfangQZLF-L-17_pos_56_01_14151.d
Bruker Compass DataAnalysis 4.1 printed: 2/14/2023 9:25:27 AM by: SCSIO Page 1 of 1

Figure S53 IR spectrum of compound **6**

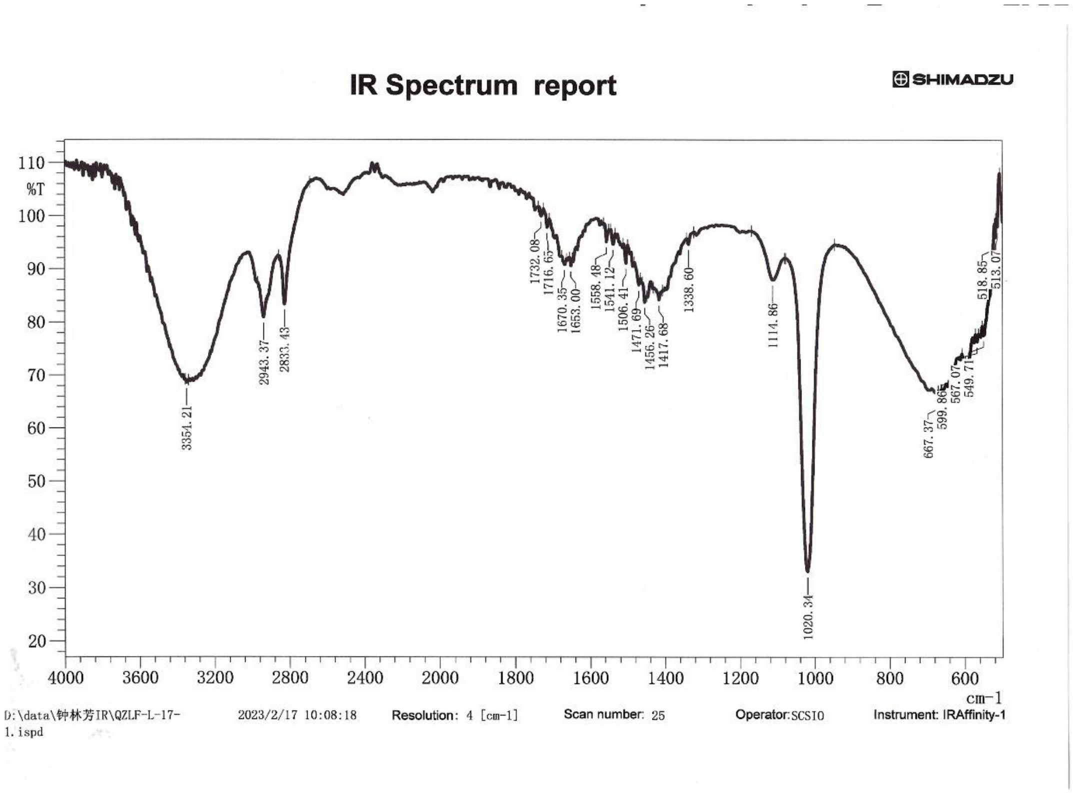

Figure S54 UV spectrum of compound **6**

光谱峰值检测报告

2023/02/17 11:38:57

数据集: QZLF-L-17 - RawData

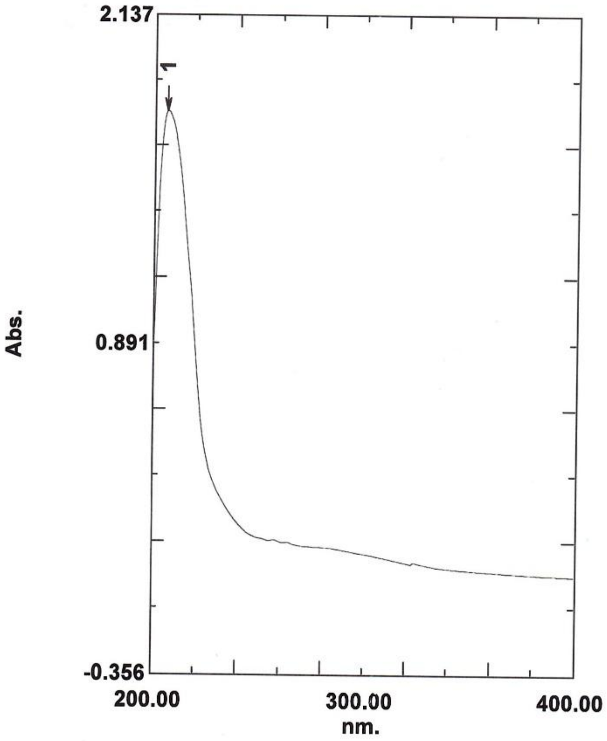

[测定属性]
波长范围(nm): 200.00 到 400.00
扫描速度: 中速
采样间隔: 0.2
自动采样间隔: 启用
扫描模式: 单个

[仪器属性]
仪器类型: UV-2600 系列
测定方式: 吸收值
狭缝宽: 2.0
积分时间: 0.1 秒.
光源转换波长: 323.0 nm
检测器单元: 直接
S/R 转换: 标准
阶梯校正: OFF

[附件属性]
附件: 无

[数据处理参数]
阈值: 0.0500000
点: 4
内插: 停用
平均: 停用

[样品准备属性]
重量:
体积:
稀释:
光程长:
附加信息:

No.	P/V	波长(nm)	Abs.	描述
1	⬆	206.00	1.769	

页 1/1

Figure S55 X-ray crystallographic structure of compound **3**

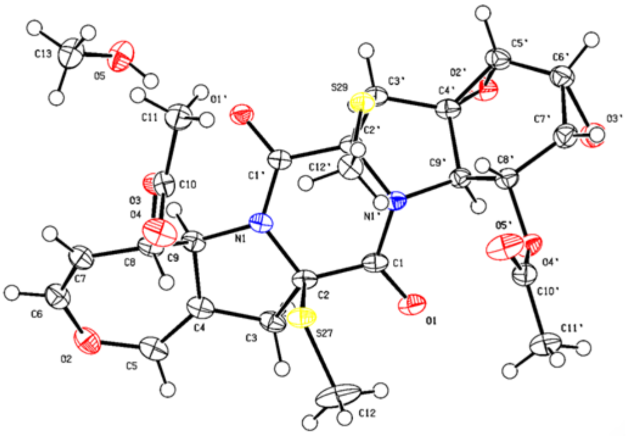

3

Table S1. Crystal Data and Structure Refinement for Compound **3**

Identification code	QZLF-L-13_collect
Empirical formula	$C_{25}H_{30}N_2O_{10}S_2$
Formula weight	582.63
Temperature/K	100.00(10)
Crystal system	monoclinic
Space group	$P2_1$
a/Å	7.14328(5)
b/Å	16.25526(10)
c/Å	11.41675(7)
α/°	90
β/°	93.4877(6)
γ/°	90
Volume/Å^3	1323.211(14)
Z	2
ρ_{calc}g/cm^3	1.462
μ/mm^{-1}	2.357
F(000)	612.0
Crystal size/mm^3	0.15 × 0.12 × 0.1
Radiation	Cu Kα (λ = 1.54184)
2Θ range for data collection/ °	7.758 to 148.752
Index ranges	-8 ≤ h ≤ 8, -19 ≤ k ≤ 20, -14 ≤ l ≤ 14
Reflections collected	25678

Independent reflections	5292 [R_{int} = 0.0323, R_{sigma} = 0.0216]
Data/restraints/parameters	5292/1/358
Goodness-of-fit on F^2	1.075
Final R indexes [I>=2σ (I)]	R_1 = 0.0242, wR_2 = 0.0639
Final R indexes [all data]	R_1 = 0.0244, wR_2 = 0.0641
Largest diff. peak/hole / e Å^{-3}	0.23/-0.17
Flack parameter	-0.001(4)

Table S2 Relative thermal energies (ΔE), relative free energies (ΔG), and equilibrium populations (P) of low-energy conformers of structures **1-6** in MeOH or MeCN

conformer	ΔE(kcal/mol)[a]	ΔG(kcal/mol)[a]	P(%)[b]	conformer	ΔE(kcal/mol)[a]	ΔG(kcal/mol)[a]	P(%)[b]
structure **1**	-	-	-	**4**-13 [c]	6.08	4.83	0.00
**1**-1	0.00	0.00	0.45	**4**-14	1.40	1.37	0.02
**1**-2	0.27	0.24	0.30	structure **5**	-	-	-
**1**-3	0.29	0.34	0.25	**5**-1	1.42	2.09	0.01
structure **2**	-	-	-	**5**-2 [c]	1.02	2.35	0.00
**2**-1	0.00	0.00	0.97	**5**-3	0.56	1.77	0.02
**2**-2	4.57	4.50	0.01	**5**-4	0.71	2.06	0.01
**2**-3	0.82	2.27	0.02	**5**-5	0.93	0.00	0.37
structure **3**	-	-	-	**5**-6 [c]	5.07	6.59	0.00
**3**-1	2.20	2.40	0.01	**5**-7	0.90	1.53	0.03
**3**-2	1.37	1.85	0.03	**5**-8 [c]	4.16	4.98	0.00
**3**-3 [c]	3.80	3.76	0.00	**5**-9	0.00	0.01	0.36
**3**-4 [c]	2.57	3.18	0.00	**5**-10	0.90	1.53	0.03
**3**-5	3.26	2.32	0.01	**5**-11	0.55	1.76	0.02
**3**-6	0.00	1.32	0.07	**5**-12	0.31	1.32	0.04
**3**-7	1.21	2.20	0.02	**5**-13 [c]	4.53	3.73	0.00
**3**-8	0.86	0.00	0.69	**5**-14	1.05	1.51	0.03
**3**-9 [c]	5.21	4.10	0.00	**5**-15	0.81	0.92	0.08
**3**-10	0.46	1.40	0.06	structure **6**	-	-	-
**3**-11 [c]	3.80	3.76	0.00	**6**-1	2.15	2.42	0.01
**3**-12	0.46	1.39	0.07	**6**-2	2.11	2.19	0.01
**3**-13	1.36	1.85	0.03	**6**-3	2.11	2.19	0.01
structure **4**	-	-	-	**6**-4 [c]	2.45	2.76	0.00
**4**-1	1.79	1.06	0.04	**6**-5 [c]	2.61	2.89	0.00
**4**-2	0.00	0.04	0.23	**6**-6	0.00	0.00	0.37
**4**-3	1.95	1.06	0.04	**6**-7	1.04	0.00	0.37
**4**-4	1.79	1.06	0.04	**6**-8	0.17	0.40	0.19
**4**-5	0.25	0.00	0.25	**6**-9	2.08	1.70	0.02

**4**-6	0.00	0.03	0.24	**6**-10 [c]	3.32	3.69	0.00
**4**-7	1.79	1.06	0.04	**6**-11 [c]	2.82	2.74	0.00
**4**-8 [c]	4.50	4.45	0.00	**6**-12	2.16	2.42	0.01
**4**-9	2.18	2.00	0.01	**6**-13 [c]	5.60	5.60	0.00
**4**-10	1.38	1.11	0.04	**6**-14 [c]	3.57	3.95	0.00
**4**-11 [c]	2.92	2.85	0.00	**6**-15 [c]	3.22	3.22	0.00
**4**-12	1.95	1.06	0.04	-	-	-	-

[a] At the M06-2X/def2-TZVP/ SMD level of theory.

[b] From ΔG values at 298.15 K.

[c] Conformer not applied to ECD/TDDFT calculations.

Figure S56 Conformations of low-energy conformers of structure **1** in MeOH

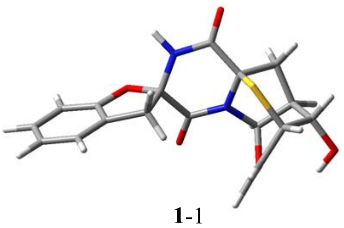

1-1

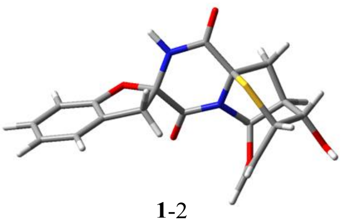

1-2

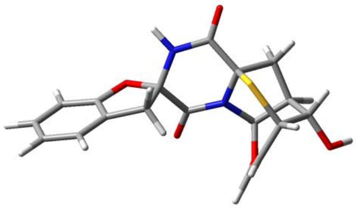

1-3

Figure S57 Conformations of low-energy conformers of structure **2** in MeOH

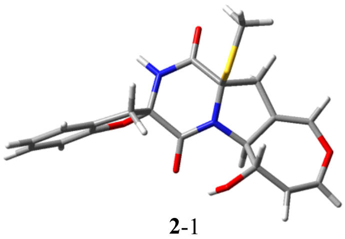

2-1

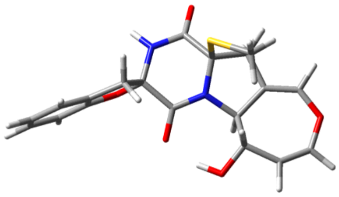

2-2

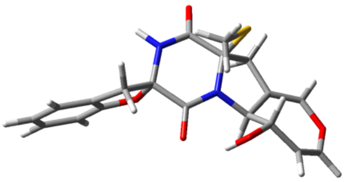

2-3

Figure S58 Conformations of low-energy conformers of structure **3** in CH_3CN

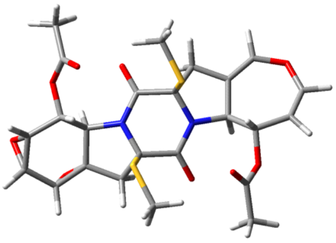

3-1

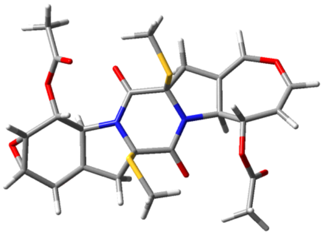

3-2

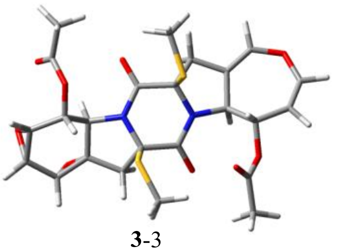

3-3

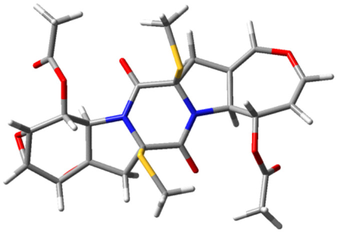

3-4

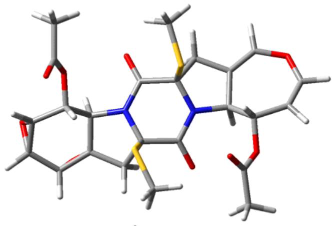

3-5

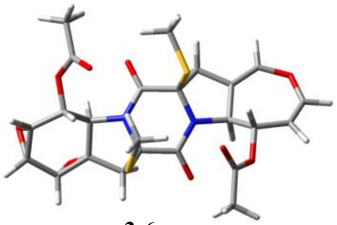

3-6

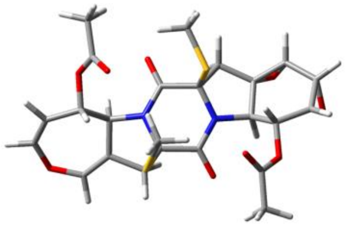

3-7

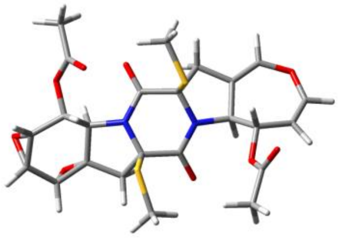

3-8

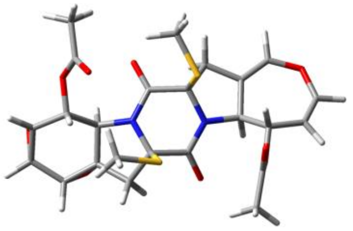

3-9

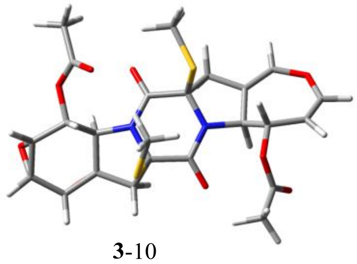

3-10

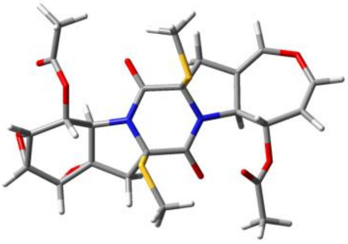

3-11

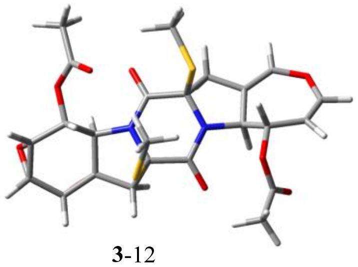

3-12

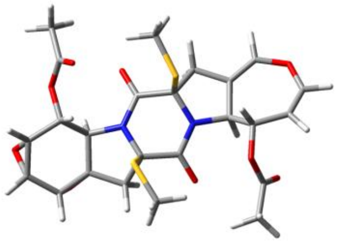

3-13

Figure S59 Conformations of low-energy conformers of structure **4** in MeOH

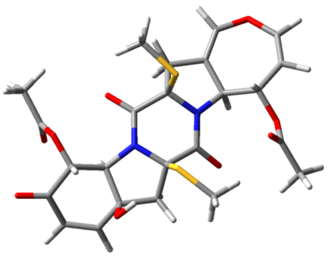

4-1

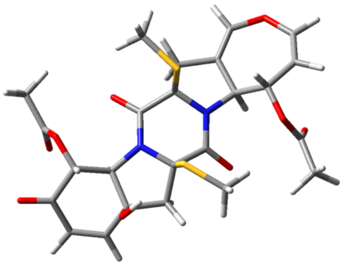

4-2

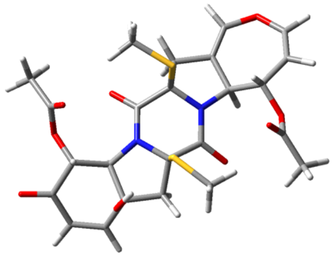

4-3

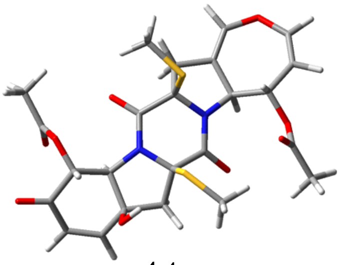

4-4

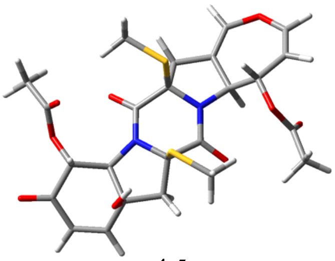

4-5

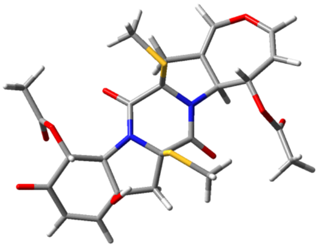

4-6

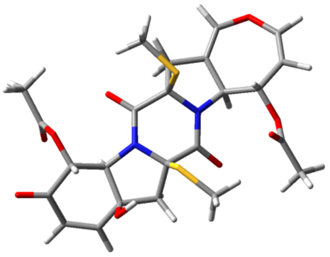

4-7

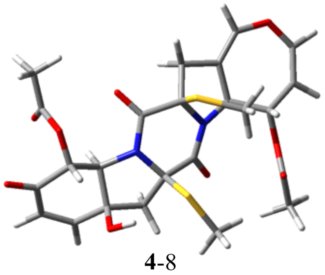

4-8

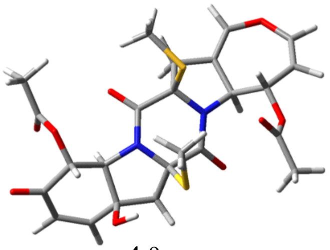

4-9

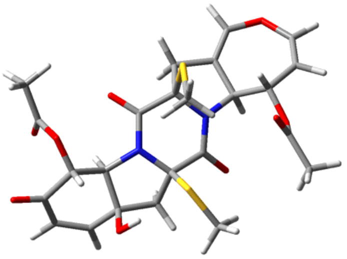

4-10

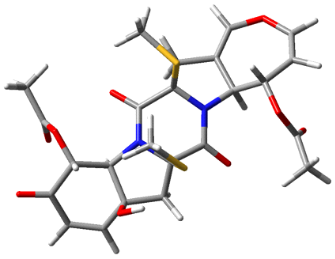

4-11

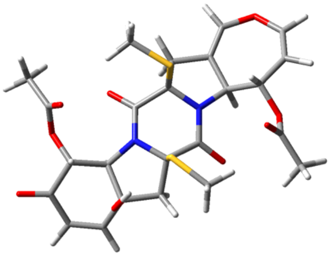

4-12

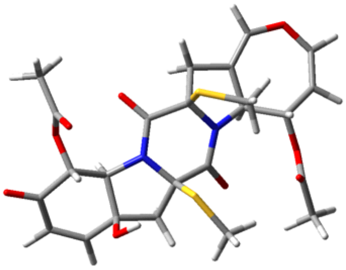

4-13

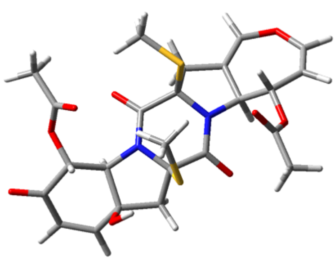

4-14

Figure S60 Conformations of low-energy conformers of structure **5** in MeOH

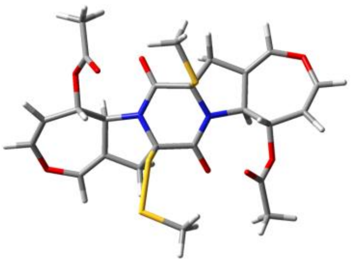

5-1

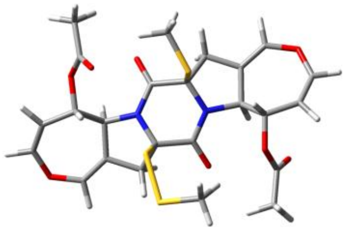

5-2

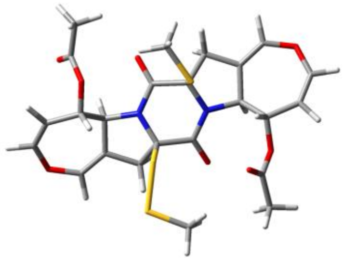

5-3

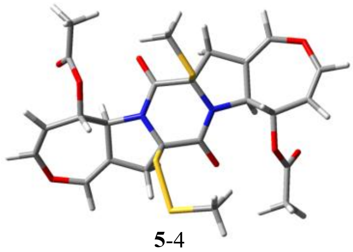

5-4

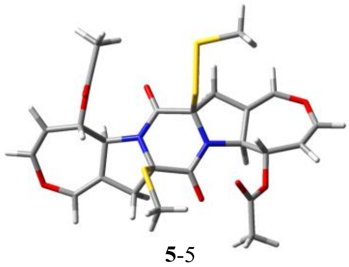

5-5

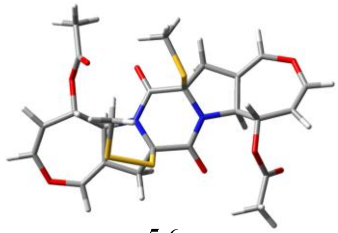

5-6

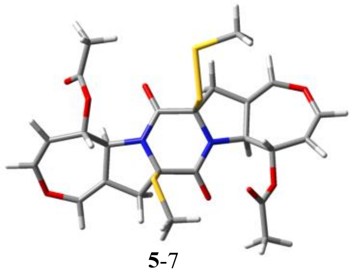

5-7

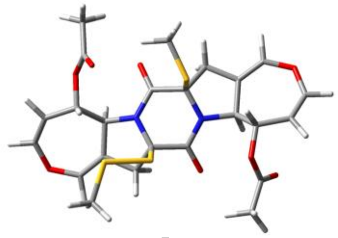

5-8

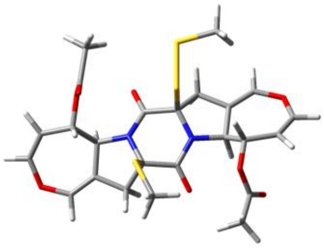

5-9

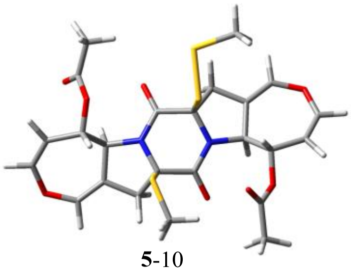

5-10

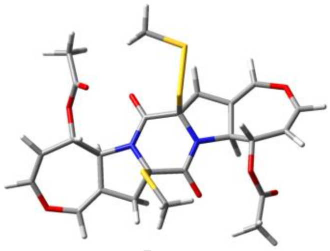

5-11

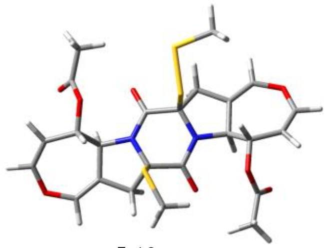

5-12

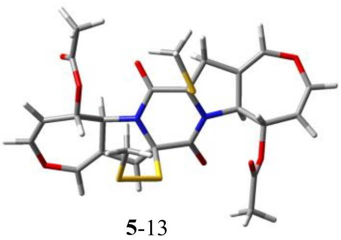

5-13

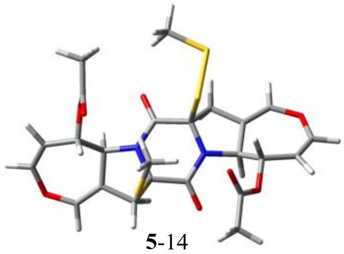

5-14

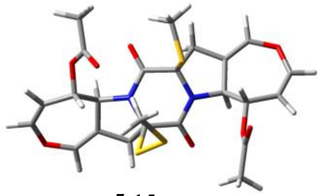

5-15

Figure S61 Conformations of low-energy conformers of structure **6** in MeOH

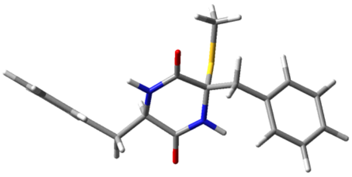

6-1

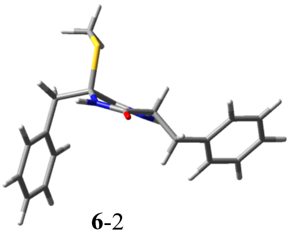

6-2

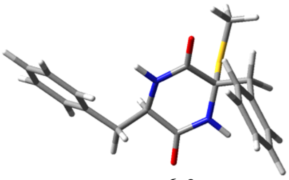

6-3

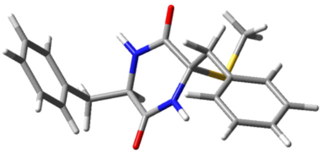

6-4

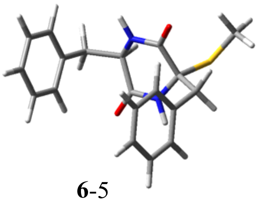

6-5

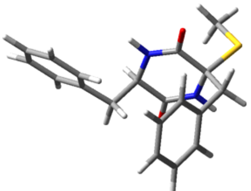

6-6

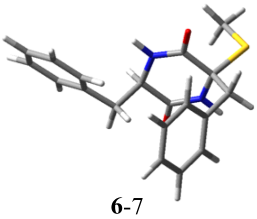

6-7

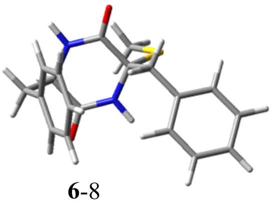

6-8

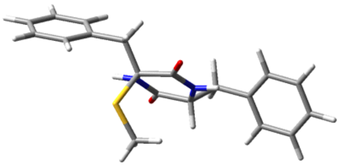

6-9

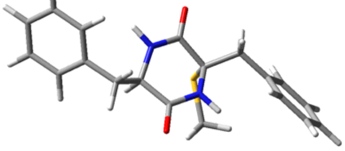

6-10

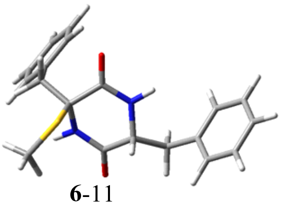

6-11

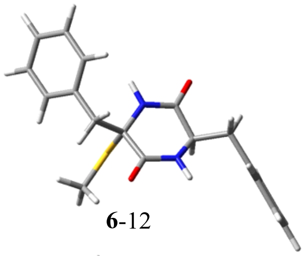

6-12

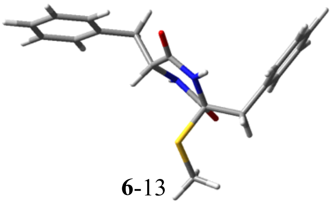

6-13

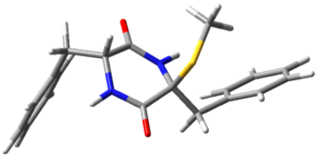

6-14

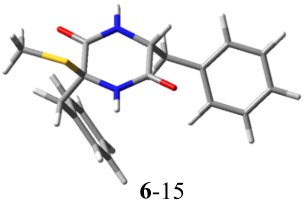

6-15

Figure S62 CD spectrum of **3** in CH_3CN

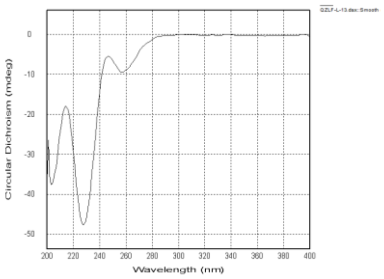

3

Figure S63 CD spectra of **1, 2**, **4-6** in MeOH

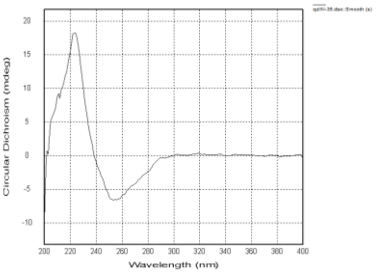

1

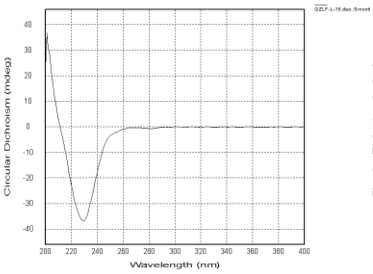

2

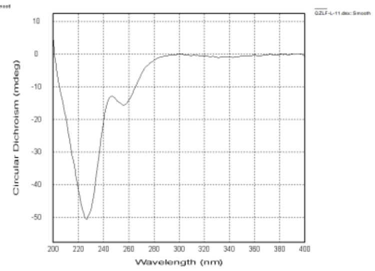

4

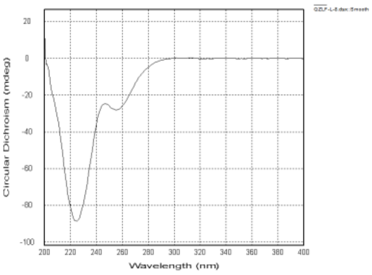

5

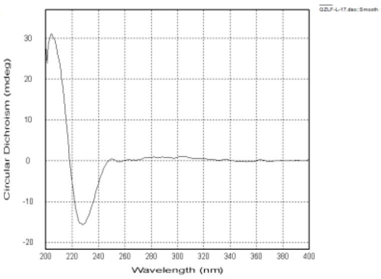

6

Figure S64 Key HMBC and COSY correlations of compounds **5-6**

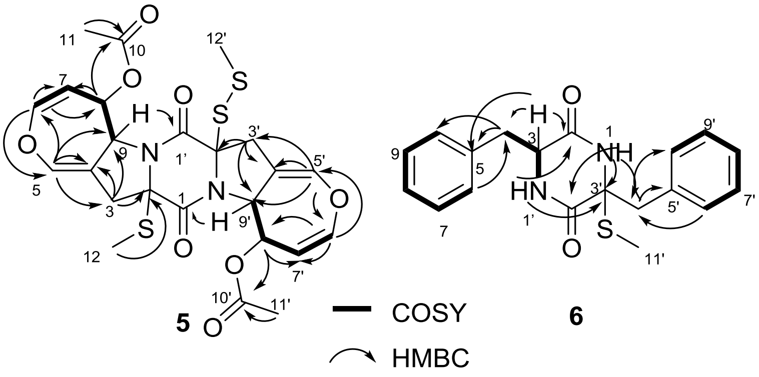

Figure S65 Key NOESY correlations of compounds **5-6**

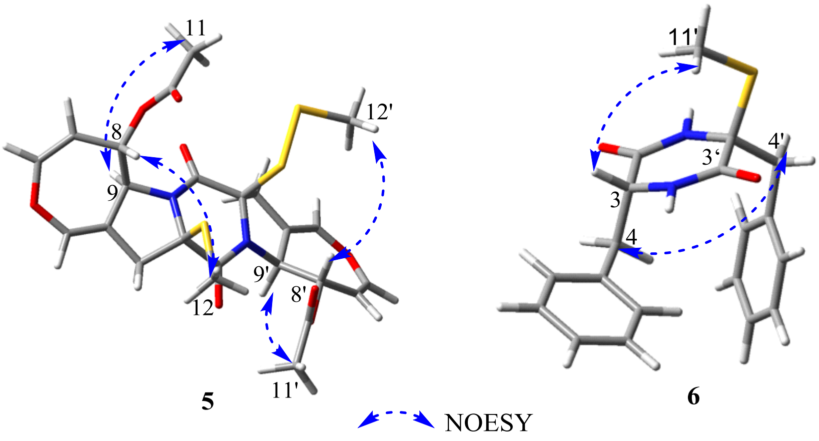

Figure S66 The proposed biosynthetic pathway for compound **1**

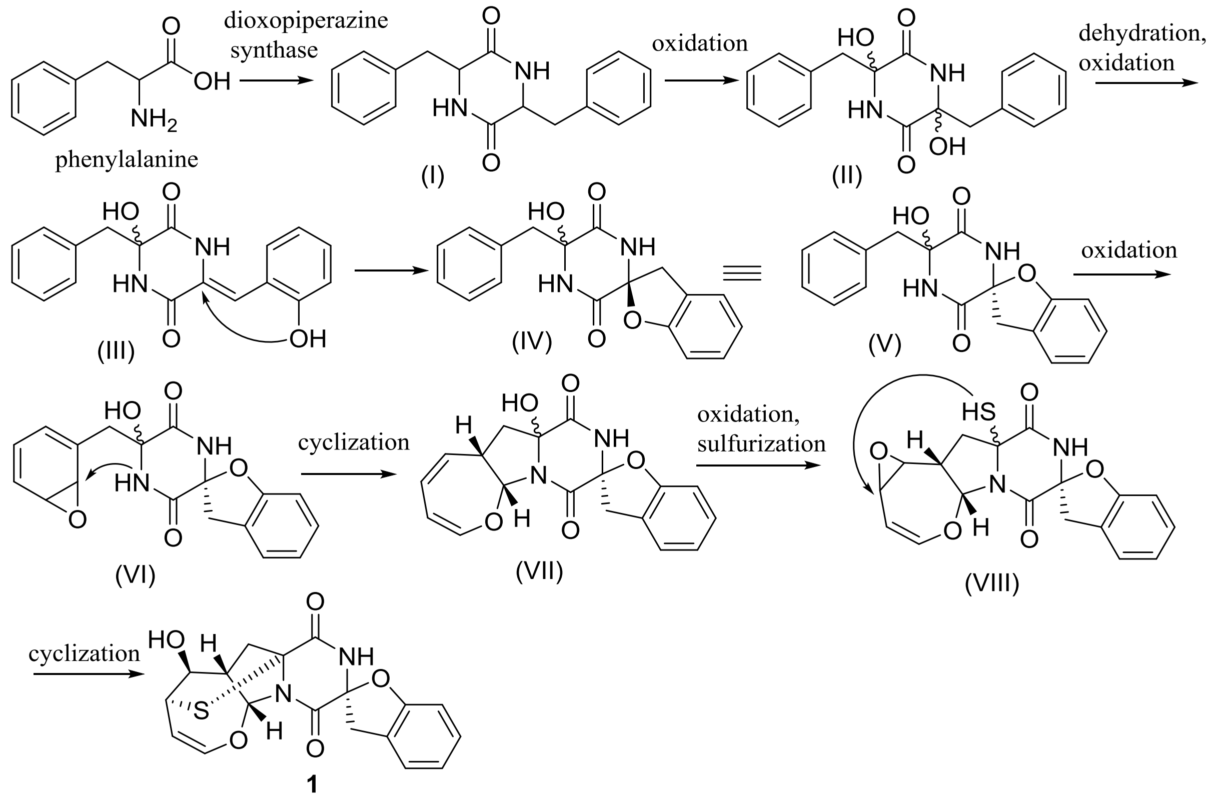

Supplement: Supplementary file 1 [file marinedrugs-21-00575-s001.zip › marinedrugs-2669183-supplementary.pdf]
